# Integrating Dense Genotyping with High‐Throughput Phenotyping Empowers the Genetic Dissection of Berry Quality and Resilience Traits in Grapevine

**DOI:** 10.1002/advs.202412587

**Published:** 2025-05-08

**Authors:** Yuyu Zhang, Yongjian Wang, Michael Henke, Pablo Carbonell‐Bejerano, Zemin Wang, Pierre‐François Bert, Yi Wang, Huayang Li, Junhua Kong, Peige Fan, Zhanwu Dai, Zhenchang Liang

**Affiliations:** ^1^ State Key Laboratory of Plant Diversity and Specialty Crops and Beijing Key Laboratory of Grape Science and Enology Institute of Botany Chinese Academy of Sciences Beijing 100093 China; ^2^ China National Botanical Garden Beijing 100093 China; ^3^ University of Chinese Academy of Sciences Beijing 100049 China; ^4^ Instituto de Ciencias de la Vid y del Vino (ICVV) Consejo Superior de Investigaciones Científicas‐Universidad de La Rioja‐Gobierno de La Rioja Logrono 26007 Spain; ^5^ EGFV, Bordeaux Sciences Agro INRAE, ISVV, Univ. Bordeaux Villenave d'Ornon 33882 France; ^6^ Present address: School of Agronomy Hunan Agricultural University Changsha 410128 China

**Keywords:** 200K axiom SNP array, association mapping, fine mapping, haplotype analysis, high‐throughput phenotyping, NAC08

## Abstract

Investigating the genetic architecture of important agronomic traits in grapevine, like berry quality and resilience to abiotic stress, has been hampered by bottlenecks in genotyping and phenotyping. To address these limitations, this study aimed to develop innovative tools to unravel the complex polygenic genomic architecture of these traits. Specifically, a high‐density 200K single nucleotide polymorphism array is developed and validated its effectiveness by genotyping 471 accessions from three F_1_ breeding populations. A high‐throughput grape phenotyping tool is developed to accurately capture berry color, shape, and size. By integrating data from the two platforms, associated loci are identified over three growing seasons. Association mapping and haplotype analysis identified novel loci and candidate genes for berry shape (*bHLH017*), soluble sugars (*ACT*), and organic acids (*ALMT1* and *FUSC2*), as well as vine cold tolerance (*NAC08*), and fine‐mapped the flower sex determination locus. Furthermore, the functional role of NAC08 is validated, demonstrating that it activates the expression of a raffinose synthase gene, thereby increasing raffinose levels and conferring cold tolerance. Together, these augmented tools, the integrated data, and novel loci establish a better foundation for trait aggregation that will enhance breeding efficiency and boost the development of high‐quality grape varieties.

## Graphical abstract

Researchers develop advanced tools to study grapevine traits like berry quality and stress resilience. A 200K SNP array and high‐throughput phenotyping enable the identification of loci linked to berry shape, sugar content, acidity, and cold tolerance. Functional validation of genes such as *NAC08* reveals roles in cold tolerance. These innovations improve breeding efficiency for high‐quality grapes.

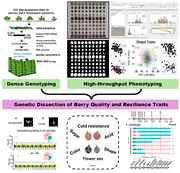

## 1 Introduction

Grapevine (*Vitis* spp.) is a highly valuable fruit crop consumed globally in diverse forms, including wine, spirits, table grapes, and raisins. Climate change poses numerous challenges to grapevine cultivation, including higher temperatures that affect grape quality by increasing sugar concentration, lowering acidity, and compromising anthocyanin content.^[^ ^1^, ^2^, ^3^ ^]^ One strategy to address these challenges is to breed high‐quality, resilient grape varieties that can adapt to climate change.^[^ ^4^, ^5^, ^6^, ^7^ ^]^ The juvenile phase of grapevine development lasts several years so it is preferable to use reliable molecular markers to accelerate breeding and streamline selection. Previous marker‐based studies have focused on individual traits like disease resistance and grape pigmentation.^[^ ^8^, ^9^, ^10^ ^]^ The genetic determinism of many desired traits in grape breeding is complex and influenced by both genotype and environmental interactions. To study such quantitative traits or to combine multiple traits in one cultivar requires extensive and precise genotypic and phenotypic data. We aimed to integrate data from molecular markers and high‐throughput phenotyping with a view to expediting the development of superior grape varieties.

Three breeding strategies take advantage of molecular markers: marker‐assisted selection,^[^ ^11^ ^]^ marker‐assisted introgression, and genomic selection.^[^ ^12^ ^]^ Marker‐assisted selection associates markers with genes or traits of interest and has been successful in breeding various plant species.^[^ ^11^ ^]^ Next‐generation sequencing technologies have enabled high‐throughput genotyping approaches, like genotyping‐by‐sequencing^[^ ^13^ ^]^ and whole genome re‐sequencing, which are used to create linkage genetic maps incorporating numerous molecular markers. However, using hybridization chips for genotyping is more straightforward to analyze than genotyping‐by‐sequencing and the reproducibility of results is higher.^[^ ^14^, ^15^, ^16^ ^]^ Single nucleotide polymorphism (SNP) chips have already been designed for several perennial woody plants, including pear,^[^ ^17^ ^]^ tea,^[^ ^18^ ^]^ and apple.^[^ ^19^ ^]^ In the case of grapevine, the initial *Vitis* 9K SNP chip^[^ ^20^ ^]^ was created using resequencing data from 6 wild *Vitis* species and 11 *Vitis vinifera* cultivars. The subsequent *Vitis* 18K SNP chip,^[^ ^21^ ^]^ collaboratively developed by the GrapeReSeq Consortium, encompassed 18,071 SNPs from different *V. vinifera* cultivars, wild species, and *Muscadinia rotundifolia* accessions. Later, to enhance marker transferability across grapevine species, 2,000 rhAmpSeq core genome markers^[^ ^15^ ^]^ were designed by aligning 10 independent *de novo* grapevine assemblies. However, the density of currently accessible SNP arrays is not sufficient for fine‐mapping complex traits.

Phenotyping is lagging behind the progress in genotyping and has become a bottleneck for genetic studies of complex traits.^[^ ^22^ ^]^ Obtaining comprehensive, precise, and reliable phenotypic data from grapevine is challenging due to the number and diversity of biological attributes and environmental interactions. High‐throughput phenotyping (HTP) based on image analysis has been successfully applied in annual crops^[^ ^23^, ^24^, ^25^ ^]^ but is more complex in perennial fruit trees like grapevine.^[^ ^26^, ^27^ ^]^ Robust HTP is required in grapes to objectively quantify multiple traits and standardize results across diverse genotypes.

We are working under the hypothesis that exploring domesticated vs. wild *Vitis spp*. hybrid populations could increase the chances to decreased polygeny for certain traits, such as cold tolerance and organic acid content, whose major effect alleles might present in the wild genotypes. To address this hypothesis, we screened 174,464 grapevine SNPs^[^ ^28^ ^]^ and incorporated them as probes in a 200K SNP array for pertinent genetic architecture identification. The effectiveness and applicability of this array were evaluated by genotyping three F_1_ *Vitis sp*. breeding populations. We further developed a cost‐effective high‐throughput phenotyping facility and utilized it to evaluate berry color and morphometry traits over three years. In parallel, we assessed other important traits with conventional phenotyping approaches, including berry content of sugars, organic acids and anthocyanins, cold tolerance of winter buds, and flower sex over multiple years. The integrated data was investigated by association mapping, fine mapping, haplotype analysis, and additive effect analysis to elucidate the genetic basis of these traits. Furthermore, we showed that one gene, *VaNAC08*, that was associated with cold tolerance, enhances the cold resistance of grapes by activating the expression of the raffinose synthase gene *VaRFS6* to increase the content of raffinose under cold stress.

## 2 Results

### 2.1 Design and Distribution of the Grapevine 200K Axiom SNP Array

**Figure 1:**
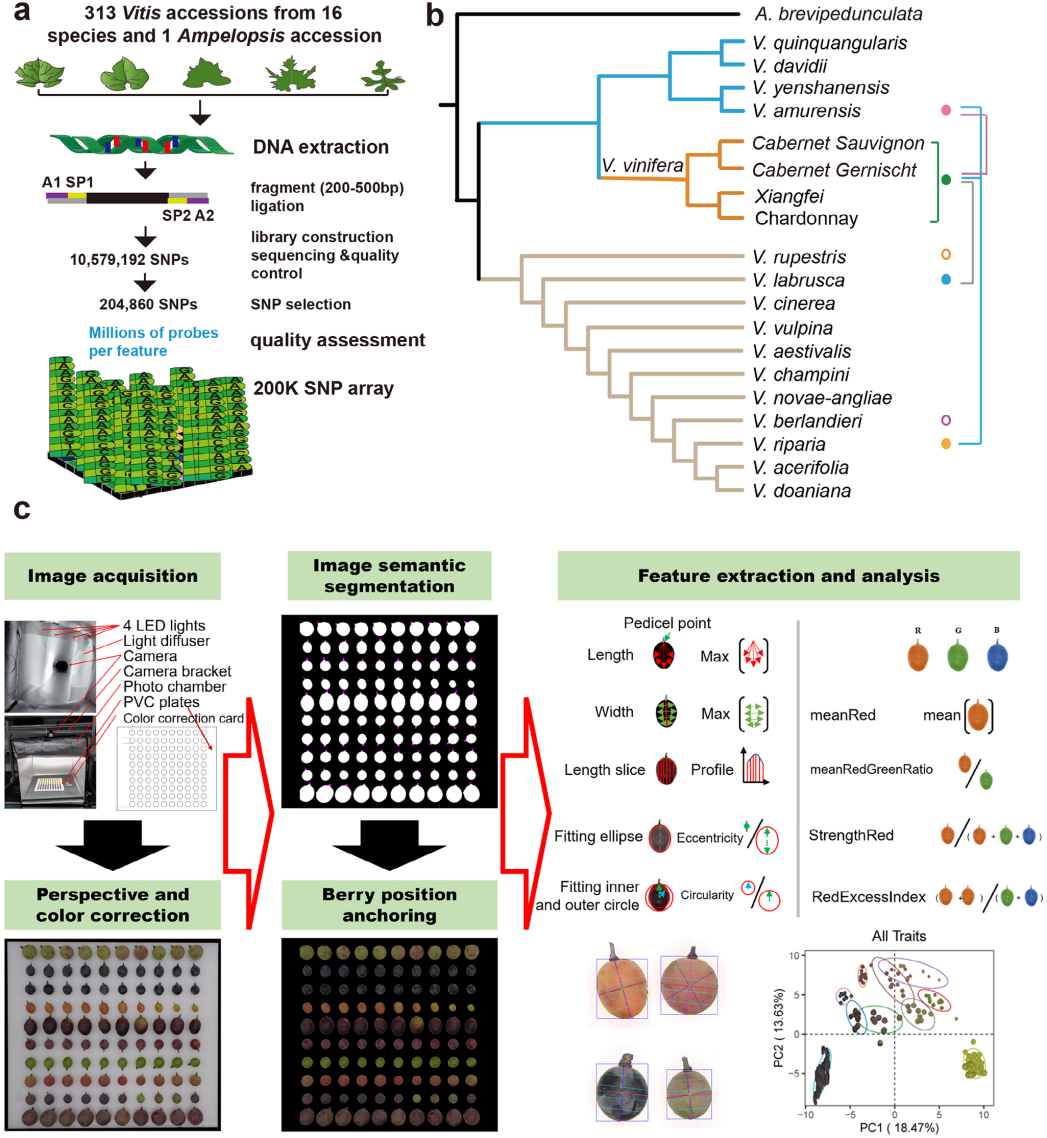
Design and construction of the grapevine 200K Axiom SNP array and schematic overview of the grape berry high‐throughput phenotyping (HTP) system. a). Key steps in designing and developing the 200K Axiom SNP array. b). Simplified evolutionary/selection relationships between 313 *Vitis* accessions from 16 species and 1 *Ampelopsis brevipedunculata* used for choosing sequences for SNPs. The maximum‐likelihood phylogenetic tree was constructed using SNPphylo.^[^ ^54^ ^]^ Two open circles denote a few interspecific genotypes which are hybrids of the two indicated species. Closed circles connected by lines denote a large number of interspecific genotypes that are hybrids of two indicated species. c). Image acquisition facility and image processing pipeline for the HTP system. i) The image acquisition facility was composed of a CCD camera (Sony ILCE‐6400), a custom plastic plate for fixing berry position, and a photo chamber consisting of lights with light‐emitting diodes, a X‐rite Color Checker classic mini card for color correction and camera support frame. ii) Perspective correction based on the background plate after image segmentation. iii) Color correction based on the X‐rite Color Checker classic mini card. iv) Semantic segmentation of berry pedicel image based on morphological operators. v) Features related to berry color, shape, and size were extracted after pedicel position correction and data analysis.

To develop and design a grapevine SNP array (200K) that contains ten times more SNPs than the current ones (e.g. 18K SNP chip^[^ ^21^ ^]^ or 2K rhAmpSeq markers^[^ ^15^ ^]^), SNPs were identified and screened from the re‐sequencing data of a panel of 313 *Vitis* genotypes and one genotype of *Ampelopsis*.^[^ ^28^ ^]^ This panel comprised 197 Eurasian accessions (*V. vinifera*), 21 accessions of *Vitis* species from North America and 12 from East Asia, 83 genotypes from interspecific‐hybrid grapevine cultivars (HYB), and the wild relative *Ampelopsis brevipedunculata* (**Figure** **1**a,b; Table S1, Supporting Information). The sequencing reads were mapped to the *V. vinifera* reference genome PN40024 12X.v0 to identify SNPs.^[^ ^29^ ^]^ A total of 10.6 million high‐quality variants were initially detected across the grape genomes, but after several iterations of quality verification and filtering only 500,724 SNPs were retained and used to prepare the 200K SNP array. After a final selection of 204,860 SNPs (further details in the Methods section and Figure 1a), a total of 174,464 SNPs were incorporated in an Axiom array. These SNPs were submitted to Affymetrix (now part of Thermo Fisher Scientific, USA) and successfully tiled on a chip to produce the grapevine Axiom 200K SNP array.

**Figure 2:**
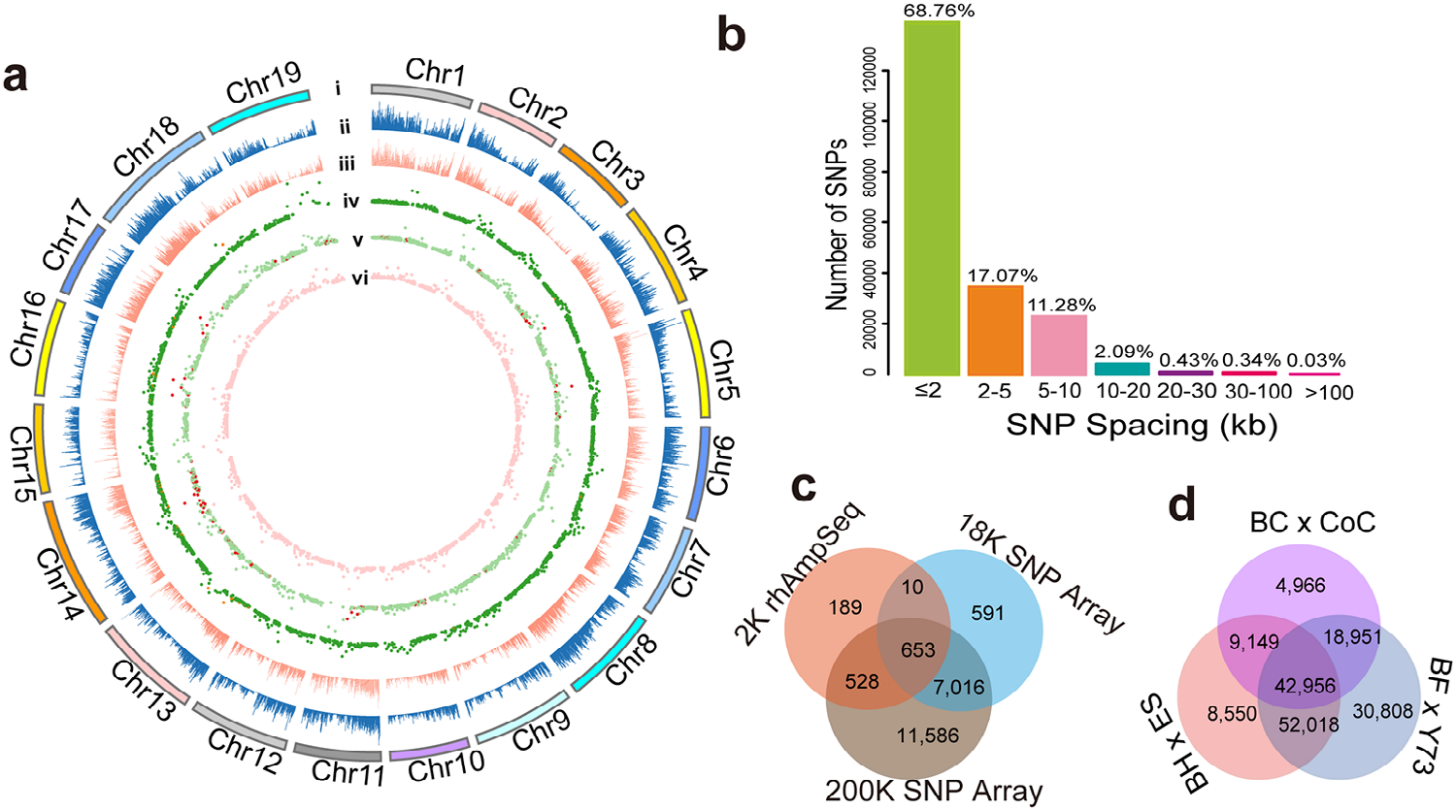
Performance of 200K SNP array markers. a) Genomic view of performance of different types of SNP markers. i) Circular representation of the 19 chromosomes of the grapevine genome. Physical locations showing relative density with a 100 kb sliding window of ii) all 200K SNP markers or iii) only polymorphic high‐resolution and no‐minor‐homozygosity markers. (iv‐vi) The positions of SNP markers in the linkage maps of the F_1_ breeding populations iv) BF × Y73, v) BH × ES, and vi) BC × CoC are indicated by dark green, light green, and pink dots, with red dots representing markers showing biased segregation. b) The distribution of inter‐SNP spacing, measured in kilobases (kb). c) The number of genes covered by SNP markers from previously reported grapevine 18K SNP array^21^ and 2K rhAmpseq SNPs^15^ compared to the current 200K SNP array. d) Venn diagram of the number of SNPs in the three F_1_ populations. The high number of SNPs common to two or three populations indicates the robustness of the 200K SNP array for genotyping cross‐breeding populations.

The effects of all 174,464 SNPs were annotated successfully using the SnpEff software (version 3.6c)^[^ ^30^ ^]^ and ‘Bartlett’ v1.0. According to the annotation, 16,093 SNPs (9.2%) were classified as non‐synonymous polymorphisms with ‘High’ and ‘Moderate’ impacts on the phenotype, while 26,666 SNPs (15.3%) were considered to have a ‘Low’ impact. A further 131,705 SNPs (75.5%), the majority, were categorized as ‘Modifier’ type (Table S2, Supporting Information). These 174,464 SNPs are uniformly distributed along the 19 chromosomes of the grapevine reference genome, except for those located on an unknown chromosome (**Figure** **2**a). Hence, the estimated grape anchored genome assembly exhibits an average distribution of approximately one SNP per 2.8 kb.^[^ ^29^ ^]^ Most of these SNPs (97.11%) are spaced at 10 kb or less from each other, including 68.76% spaced at 2 kb or less, and 17.07% spaced within 2–5 kb of another marker (Figure 2b). This is clearly a denser distribution than previous examples of massive genotyping of grapevine.^[^ ^15^, ^21^ ^]^

The 174,464 SNPs in the 200K array collectively span the gene bodies of 19,783 genes according to PN40024 12X V2 gene annotations (Figure 2c; Tables S3 and S4, Supporting Information). By comparison, the grape 18K SNP array^[^ ^20^ ^]^ consists of 18,071 SNPs spanning 8,270 genes, while the 2K rhAmpseq marker array^[^ ^15^ ^]^ included 2,000 SNPs spanning 1,378 genes (Figure 2c; Table S4, Supporting Information). Importantly, the genes covered by the 200K SNP array represent 85.6% of the genes covered by the 2K rhAmpseq markers and 92.7% of the genes covered by the 18K SNP array. Due to the higher density and extensive coverage, the current 200K SNP array is more representative than previous arrays with substantial overlaps to allow anchoring and cross‐referencing (Figure 2c; Figure S1, Supporting Information).

### 2.2 Grapevine Population Genotyping and Population Structure

A comprehensive set of 471 *Vitis* accessions (Table S5, Supporting Information) was genotyped using the grapevine Axiom 200K SNP array. Specifically, 6 parent cultivars (BC, CoC, BH, ES, BF, and Y73) were genotyped as well as 465 F_1_ individuals from three inter‐species hybrid populations (namely BC × CoC, BH × ES, and BF × Y73 whose pedigrees are indicated in Figure S2, Table S5, Supporting Information).

To ensure high data quality, all samples underwent a dish quality control (DQC) check, which confirmed DQC values exceeded 0.82. Ten samples had a call rate below 97%, indicating potential issues with the data, so they were excluded from further analysis. According to the quality evaluation metrics for SNPs (https://www.thermofisher.com), six classes of markers were identified (Figure S3, Table S6, Supporting Information). Approximately 54.19% of the SNPs fell into the ‘Poly High Resolution’ category, resolving well into three genotype clusters with a minimum of two instances of the minor allele (Figure S3, Supporting Information). These high‐quality markers are codominant and polymorphic SNPs. Approximately 18.92% of the SNPs were classified as ‘No Minor Homozygote’, 2.47% as ‘Off‐Target Variants’, 1.40% as ‘Mono High Resolution’, 3.91% as ‘Call Rate Below Threshold’, and 19.10% as ‘Other’ category (Figure S3, Supporting Information). It was noted that a total of 42,956 marker positions were shared between the three F_1_ populations, demonstrating consistent performance for genotyping populations originating from distinct *Vitis* species (Figure 2a,d).

**Figure 3:**
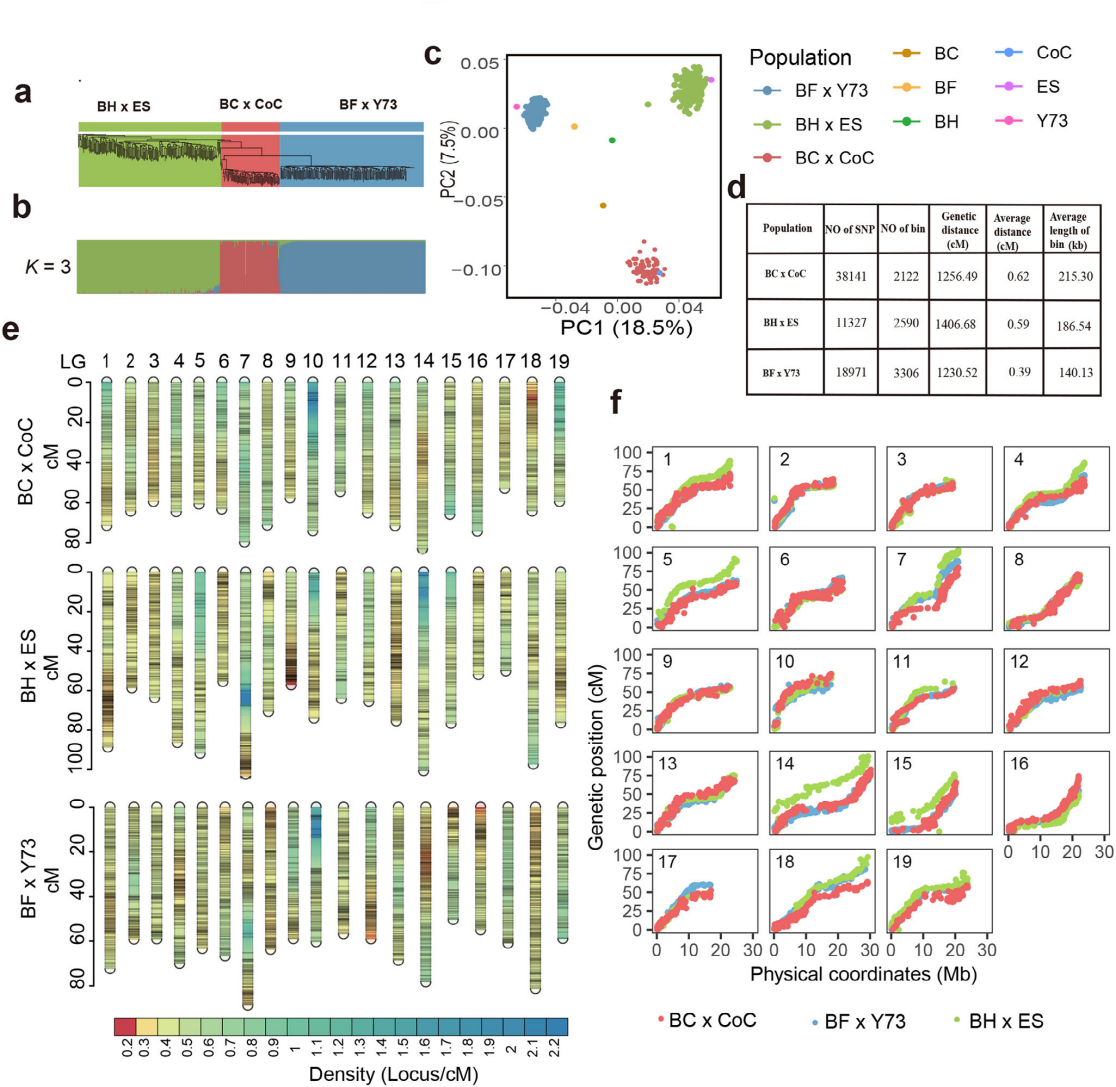
Population structure of 454 *Vitis* genotypes and the genetic maps of three breeding populations. a) The phylogenetic tree of grapevine accessions was constructed using the maximum likelihood. b) Population structure of 454 *Vitis* genotypes from three breeding populations designated as BH × ES, BC × CoC, and BF × Y73 with three clusters allowed. c) PCA plots of the first two components of variation in 454 *Vitis* genotypes relative to parental genotypes. d) Characteristics of the genetic maps of the three F_1_ populations. e) Genetic maps of three F_1_ populations. f) The relationship between the genetic position and physical location for the nineteen chromosomes of the three F_1_ populations. Genetic position, centimorgan (cM); Physical coordinates, Mb.

To explore the genetic diversity, relatedness, and clustering of diverse grape cultivars, various analytical techniques were employed, including population structure analysis using ADMIXTURE,^[^ ^32^ ^]^ the construction of a neighbor‐joining tree, and principal component analysis (PCA). The analysis revealed that each inter‐species hybrid population forms a distinct clade (**Figure** **3**a). According to the results of the population structure analysis when only two clusters (*K* = 2) were allowed, the studied genotypes fell into two main groups: the BF × Y73 hybrid F_1_ progenies and the BH × ES hybrid F_1_ progenies (Figure 3b), as expected for *V. vinifera* and wild *Vitis ssp*. or hybrid genetic backgrounds (Figure S2, Supporting Information), respectively. The observation of this division was corroborated by the PCA results (Figure 3c). When three defined clusters (*K* = 3) were allowed, the BC × CoC groups separated from the BH × ES hybrid F_1_ progenies according to the second principal component of variance (Figure 3b,c). In summary, the analysis of population genetic diversity revealed the presence of distinct clades for the three populations (Figure 3b; Figure S4, Supporting Information), attesting to the efficacy of the 200K SNP array.

### 2.3 High‐Density Genetic Linkage Map Construction

The genetic mapping framework was constructed for all three populations. For the BF × Y73 population, as many as 161,874 reliable SNP markers were detected. After filtering and merging adjacent markers with the same genotype, the ultimate genetic linkage map comprised 3,306 bin markers, which represent 18,971 SNPs (Figure 3d,e; Tables S7 and S8a, Supporting Information). The cumulative genetic distance encompassed by the bin map was 1,230.52 cM, with an average interval of 0.39 cM between neighboring bin markers. The physical length of the bin markers varied from 95.26 to 214.48 kb, with an average length of 140.13 kb (Table S8a, Supporting Information).

Using the same criteria, a cumulative count of 144,732 SNP markers of exceptional quality were detected and filtered for the BH × ES population, while 154,364 were identified for the BC × CoC population. After filtering, 11,327 SNPs were then chosen to create the consensus genetic map for the BH × ES population, which consisted of 2,590 bin markers and covered a cumulative genetic distance of 1,406.68 cM, with an average interval of 0.59 cM between neighboring bin markers (Figure 3d,e; Tables S7 and S8b, Supporting Information). Similarly, 38,141 SNPs were utilized to create the consensus genetic maps for the BC × CoC population, which consisted of 2,122 bin markers and covered a cumulative genetic span of 1,256.49 cM, with an average separation of 0.62 cM between neighboring bin markers (Figure 3d,e; Tables S7 and S8c, Supporting Information). Additionally, all three populations showed good linearity between linkage maps and physical locations, with an average Pearson's correlation coefficient spanning from 0.92 to 0.93 genome‐wide (Figure 3f).

### 2.4 Construction and Validation of an HTP Tool for Grape Berries

To efficiently screen large populations of grapes for berry color and morphometric traits, we designed a phenotyping system consisting of two main components: a high‐throughput berry image acquisition platform and an automated image processing pipeline to extract quantitative phenotypic data from images (Figure 1c).

**Figure 4:**
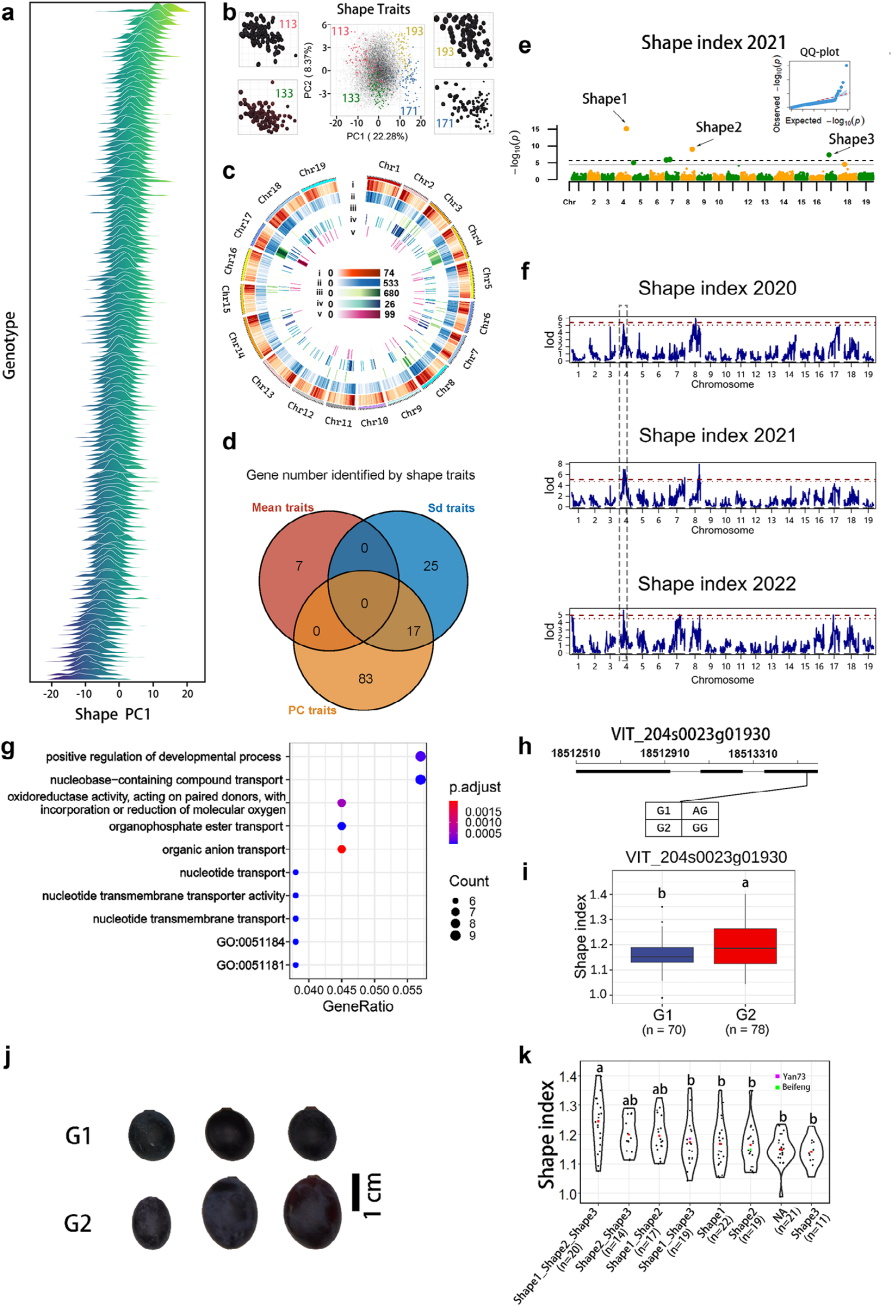
High‐throughput phenotyping and association analysis of berry shape. The mean and intra‐variation (sd) of berry shape traits were phenotyped for 100 berries per genotype at maturity from the BF × Y73 population. PCA analyses were conducted to discriminate genotypes of the population with berry shape traits. a) The distribution of PC1 for each genotype of the population was shown. b) The berry images of extreme genotypes (their genotype number indicated) identified with PCA were projected into the PCA scattering space to visualize their phenotypes. c) The density profile of significant SNPs identified by GWAS for mean, intra‐variation (sd), and composite (PC) traits. The concentric circles from outer to inner represent gene density i), SNP density ii), mean traits SNP density iii), intra‐variation (sd) traits SNP density iv), composite (PC) traits SNP density v). Different color bars indicate the range of each density. The reference genome is PN40024 12X.v0. d) Venn diagram of the number of genes identified by mean, intra‐variation (sd), and composite (PC) traits. e) Manhattan plot for shape index in 2021. The GWAS analysis was performed using BLINK. f) QTL mapping results for shape index in 2020, 2021 and 2022. g) GO analysis results of genes in Shape1 exhibiting significant haplotypic differences meanwhile containing nonsynonymous SNPs in gene CDS regions. The black dashed box in Figures e,f indicates the locus where new gene (*VIT_204s0023g01930*) identified by shape index. The structure of the candidate gene, its genotypes h) and the genotype distributions i) in the population, representative berry samples of each genotype at maturity j) and cumulative effects k) are shown. The presence of different letters above the boxplots in i,k) indicates statistically significant differences (*p* < 0.05) in the distribution of values among different genotypes, as determined through the application of Tukey's test. The sample size was shown under each boxplot in i and k.

After acquiring and processing images (Figure 1c) of individual grape berries, 124 traits are extracted related to berry size (such as length, width, and projected area), shape (such as shape index which is defined as length/width, eccentricity, and circularity), and color (such as mean values in different color spaces, color strength, and color ratios) (Figure 1c; Table S9, Supporting Information). For a single berry, all traits could be extracted in 1 s; by comparison, conventional approaches measuring far fewer berry traits take minutes to hours (Table S10, Supporting Information). The extracted traits were then used to generate composite traits from the first two principal components of variance to characterize berry color and shape through feature grouping and principal component reduction (**Figure** **4**b).

We evaluated the reliability and accuracy of the HTP system by phenotyping 14 grape cultivars exhibiting diversity in fruit color and shape (Figure S5, Supporting Information). The traits extracted by the tool were compared with those obtained through conventional manual methodologies. There is a significant positive correlation between the two ways of measuring traits (Figure S6, Supporting Information). Specifically, for the traits related to shape and size (Figure S6a–c, Supporting Information), the measurements obtained with the tool showed remarkably strong agreement (R^2^ = 0.98–0.99) with the ground‐truth assessments. For color traits, the tool performance was fairly accurate, with R^2^ values of 0.66 for the green‐to‐red spectrum and 0.84 for the blue‐to‐yellow spectrum within the Lab color spaces (Figure S6d–f, Supporting Information). It is noteworthy that genotype classification was more efficient when simultaneously considering berry shape and color than considering only shape or only color traits (Figure S7, Supporting Information). To further evaluate the robustness of the tool, we conducted tests using different camera models, image resolutions (Figure S8, Supporting Information), and varying the placement angles and orientations of berries (Figure S9, Supporting Information). Our findings indicate that the phenotype extraction was similar when images were acquired from different cameras provided the resolution exceeded 200 pixels per inch, and when berries were placed at various orientations. This demonstrates that the tool can be reliable across diverse experimental setups and conditions.

### 2.5 Identification of Candidate Genes for Traits Measured with HTP

HTP was carried out on berries produced by the BF × Y73 F_1_ population in three years (2020, 2021, and 2022). We verified the distributions of all traits in the population and conducted genome‐wide association studies (GWAS) and quantitative trait locus (QTL) analysis for 124 individual traits related to berry morphometry and color, as well as 4 composite traits (Figure 4a,d; Figure S10a–d, and Extended View Figure 1, Extended View Figure 2 and Extended View Figure 3, Supporting Information).

Four loci were consistently identified by both GWAS and QTL mapping over the three growing seasons (Figure 4c–g; Figure S10c–g, Table S11, Supporting Information), including 2 color‐related QTLs (*Color1, Color2*) and 2 shape‐related QTLs (*Shape1, Shape2*). GWAS alone identified one color‐related QTL (*Color3*) and one shape‐related QTL (*Shape3*). Interestingly, the loci *Color2* were also identified by GWAS and QTL mapping based on conventional measurement of the anthocyanin concentration by HPLC in the three years (Figure S10e–j, Supporting Information), corroborating the pertinence and reliability of the HTP traits. The locus of *Color2* is on chromosome 2 within a region from 5.2 to 18.7 Mb, which is associated with the most individual or composite color features (Figure S10e–g, Table S11, Supporting Information). This locus includes the *VvMYBA1* gene, which is widely recognized for its pivotal role in regulating anthocyanin biosynthesis.^[^ ^8^ ^]^

The berry shape‐related locus *Shape1* was detected both by GWAS and QTL mapping of the traits of berries (Figure 4e,f). Within the region from 18.3 to 20.6 Mb of chromosome 4, *Shape1* encompasses 240 genes in total, including 70 genes exhibiting significant haplotypic differences due to nonsynonymous SNPs in gene CDS regions (Table S12, Supporting Information). Gene ontology (GO) enrichment analysis of these genes showed enrichment in the GO term “positive regulation of developmental process” (Figure 4g). Genes potentially involved in shape index regulation in grape berries were identified. Notably, the gene *VIT_204s0023g01930* contained one single nonsynonymous SNP (G/A, Ala_276_ to Thr), with two major haplotypes observed (Figure 4h) in the BF × Y73 population (Figure 4i,j). This candidate gene encodes a protein (bHLH017)^[^ ^32^ ^]^ belonging to the basic helix‐loop‐helix DNA‐binding (bHLH) family transcriptional factors. A similar grape bHLH transcription factor (*VvCEB1*) on chromosome 1 is known to tightly regulate berry size.^[^ ^33^ ^]^ However, *VvCEB1* and *bHLH017* are classified into different subfamilies within the *bHLH* gene family, as they have 50% polypeptide sequence homology. We assessed the additive effects of high shape‐index alleles by comparing the shape index in offspring with various allelic combinations within the *Shape1, Shape2*, and *Shape3* regions (Figure 4k). Offspring carrying high shape‐index alleles at all three loci consistently produced berries with higher shape‐index values compared to berries with high shape‐index alleles from only one or two loci combined (Figure 4k).

### 2.6 The Genetic Architecture of Soluble Sugars and Organic Acids

The total raw soluble sugar content of BH × ES F_1_ berries harvested in 2011, 2012, and 2013 varied between 51.84 and 291.84 g L^−1^, including glucose, which ranged from 25.66 to 136.77 g L^−1^, and fructose from 26.18 to 154.77 g L^−1^ (Figure S11, Supporting Information). In the three years, total berry organic acids ranged from 3.68 to 34.65 g L^−1^ including 2.06 to 17.18 g L^−1^ tartaric acid and 0.78 to 21.32 g L^−1^ malic acid (Figure S12, Supporting Information). We conducted a GWAS of the soluble sugars trait across three years (2011, 2012, and 2013), which revealed the presence of two sugar‐related loci named *TS1* and *TS2* on chromosomes 13 and 18, respectively (Figure S13a–g, Tables S13 and S14a, Supporting Information). QTL mapping for berry soluble sugar content in the three years also detected two loci, namely *TS1* and *TS3* on chromosomes 13 and 6. As *TS1* was detected by both approaches, it is likely to be important in regulating the soluble sugars in berries (Figure S14, Table S14b, Supporting Information).

We curated a list of genes associated with *TS1* in Table S15 (Supporting Information). Twelve genes exhibit significant haplotypic differences at the *TS1* locus while containing nonsynonymous SNPs in gene CDS regions. GO enrichment analysis of these genes showed enrichment in the GO term “organelle and mitochondrial membrane pathways or processes” (Figure S13h, Supporting Information). Prominent candidate genes potentially involved in sugar content regulation in grapes were identified. Notably, the gene *VIT_213s0019g00740* contained one single nonsynonymous SNP (G/T, Arg_407_ to Trp), which was predicted to have a significant impact (−3.62) on the function of the protein.^[^ ^34^ ^]^ Two major haplotypes were observed for this gene (Figure S13i, Supporting Information), and genotype G2 berries exhibited higher levels of soluble sugar compared to the other haplotype in 2011 (Figure S13I,j, Supporting Information). *VIT_213s0019g00740* is therefore prioritized as a candidate gene due to its association with sugar content. This candidate gene encodes a protein that contains the aspartate kinase, chorismite mutase, and TyrA (prephenate dehydrogenase) domains, known as the ACT domain repeat protein. This candidate could potentially act as a suppressor in glucose signaling pathways as the homologous protein ACT domain repeat protein9 does in Arabidopsis.^[^ ^35^ ^]^ To assess the additive effects of high‐sugar alleles on total sugar content, we compared the total sugar content in offspring with combinations of different alleles located within the *TS1* and *TS2* regions (Figure S13k, Supporting Information). Offspring carrying high‐sugar alleles at both *TS1* and *TS2* consistently produced berries with more total sugar than those with high‐sugar alleles at *TS2* alone (Figure S13k, Supporting Information).

**Figure 5:**
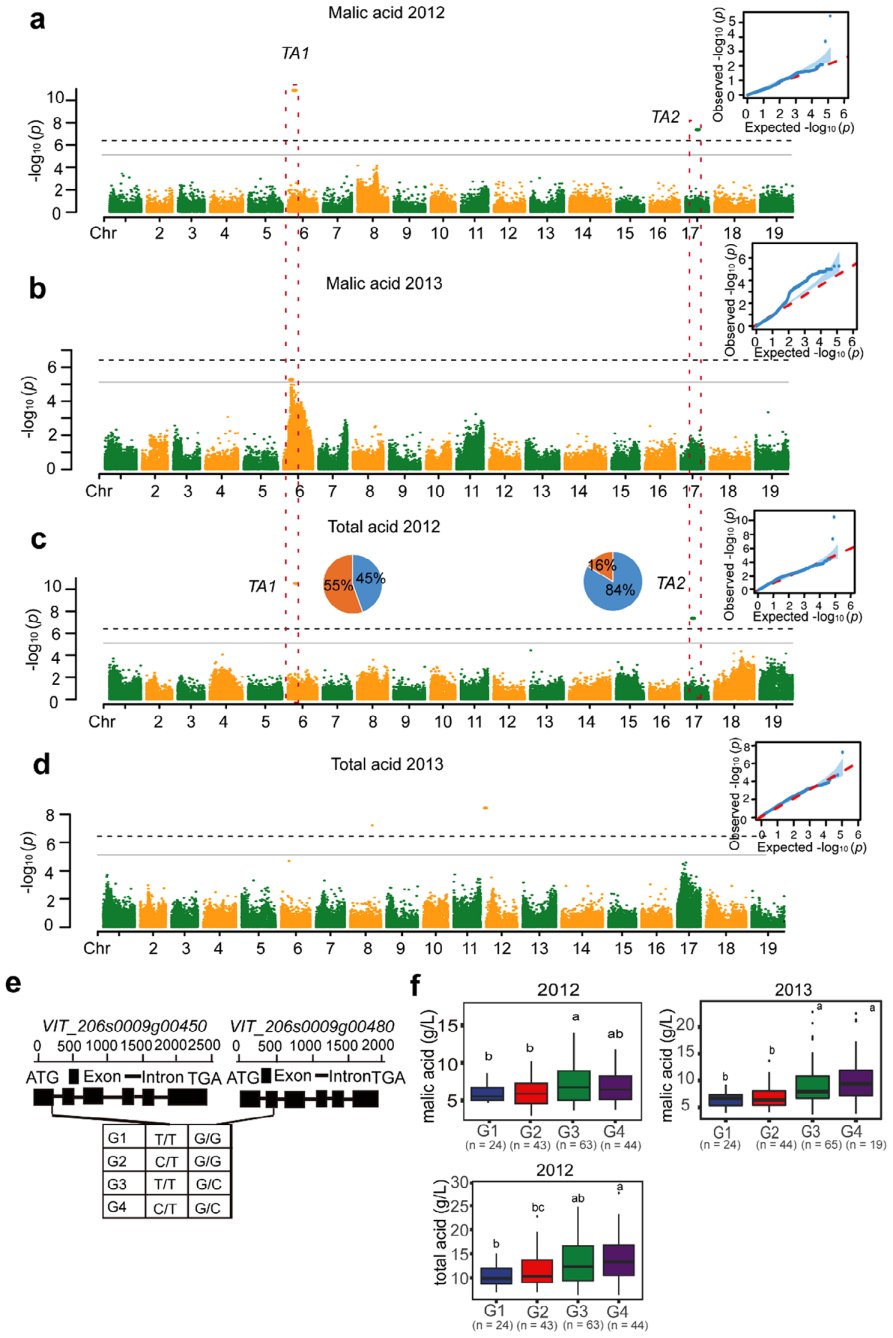
Genetic basis of berry organic acid content. a–d, Manhattan plots and QQ plots illustrating the GWAS results for organic acid content in berries within the F_1_ BH × ES population across three growing seasons. The plots display negative log_10_(*P*) values from a genome‐wide association analysis, plotted against chromosomal positions on the 19 chromosomes. The GWAS analysis was performed using BLINK. For tartaric acid, no significant loci were identified in 2011, 2012, or 2013 (data not shown). For malic acid, significant loci were identified in a) 2011 and b) 2012. For total acids, significant loci were identified in c) 2012 and d) 2013. Pie charts represent allelic frequencies of *TA1* and *TA2* loci in the populations, with the brown portion for higher total acid content and the blue portion for low total acid content. The red dashed line indicates a QTL that has been detected repeatedly. The grey line shows the significant threshold, while the black dashed line shows the highly significant threshold for *p‐*values. e) Gene structures of two candidate genes in the *TA1* locus, *VIT_206s0009g00450* encoding *ALMT1* and *VIT_206s0009g00480* encoding *FUSC2*, with haplotype results in table showing genotypes (G). f) Boxplots showing berry content of malic acid (2011) and total acid (2011, 2012, 2013) according to the four genotypes. The presence of different letters above the boxplots in f indicates statistically significant differences (*p* < 0.05) in the distribution of values among different genotypes, as determined through the application of Tukey's test. The sample size was shown under each boxplot in f).

Berry traits related to organic acid content co‐localized with a previously identified QTL for organic acid on chromosome 6^[^ ^36^ ^]^ (**Figures** **5**a–d; Figure S15, Table S14c,d, Supporting Information). The GWAS results demonstrated the association of *TA1* with malic acid content of berries in 2012 and 2013, and with total acid in berries in 2012 (Figure 5a–c; Table S14c, Supporting Information). Moreover, QTL mapping analysis confirmed an effect of *TA1* on tartaric acid in 2011 and 2013, as well as on malic acid and total organic acid in 2011, 2012, and 2013 (Figure S15, Table S14d, Supporting Information). *TA1* exhibited a high frequency of the high‐acid allele and showed a significant association with malic acid and total acid content, indicating its prevalence in the BH × ES F_1_ population. Here we detected *TA2* on chromosome 17 as a novel locus with minor frequency determining high berry acid content in this population (Figure 5a–c).

To investigate the genes that might be regulating organic acid levels, we carried out GO enrichment analysis on a set of 570 genes located within in the *TA1* locus, which are primarily associated with supramolecular polymers and supramolecular fibers (Figure S16a, Table S16, Supporting Information). We also conducted gene structure and haplotype analysis for the two known genes located within *TA1*, namely *ALMT1* (*VIT_206s0009g00450*) and *FUSC2* (*VIT_206s0009g00480*)^[^ ^37^ ^]^ (Figure 5e,f). Two SNPs were used to classify the locus into four haplotypes. We observed that genotype G4 of *ALMT1* and *FUSC2* frequently exhibited significantly higher mean levels of malic acid and total acid compared to the other haplotypes. On the other hand, the variant in *ALMT1* was synonymous. These findings suggest potential roles for *FUSC2* in influencing the levels of malic acid and total acid in grapes. To evaluate whether there is a pyramiding effect of high‐acid alleles for organic acid content, we compared total acid content among progeny carrying multiple high‐acid allelic combinations. As expected, the progeny carrying both high‐acid alleles of *TA1* and *TA2* contained more total acids (Figure S16b–d, Supporting Information).

We also explored whether there is any trait aggregation of high sugar and high total acids within the BH × ES population by assessing allelic combinations for these two traits. The results (Figure S17, Supporting Information) indicate a negative correlation trend between total sugar and total acid proportion in the population variation. Some individuals have a combination of favorable high‐acid alleles of *TA1, TA2*, and high‐sugar *TS2*, and the berries contain more sugars and total acids than the average value of the population. However, none of the study population possessed all alleles for high total sugar and total acid (Figure S17, Supporting Information).

### 2.7 The Genetic Architecture of Cold Tolerance

**Figure 6:**
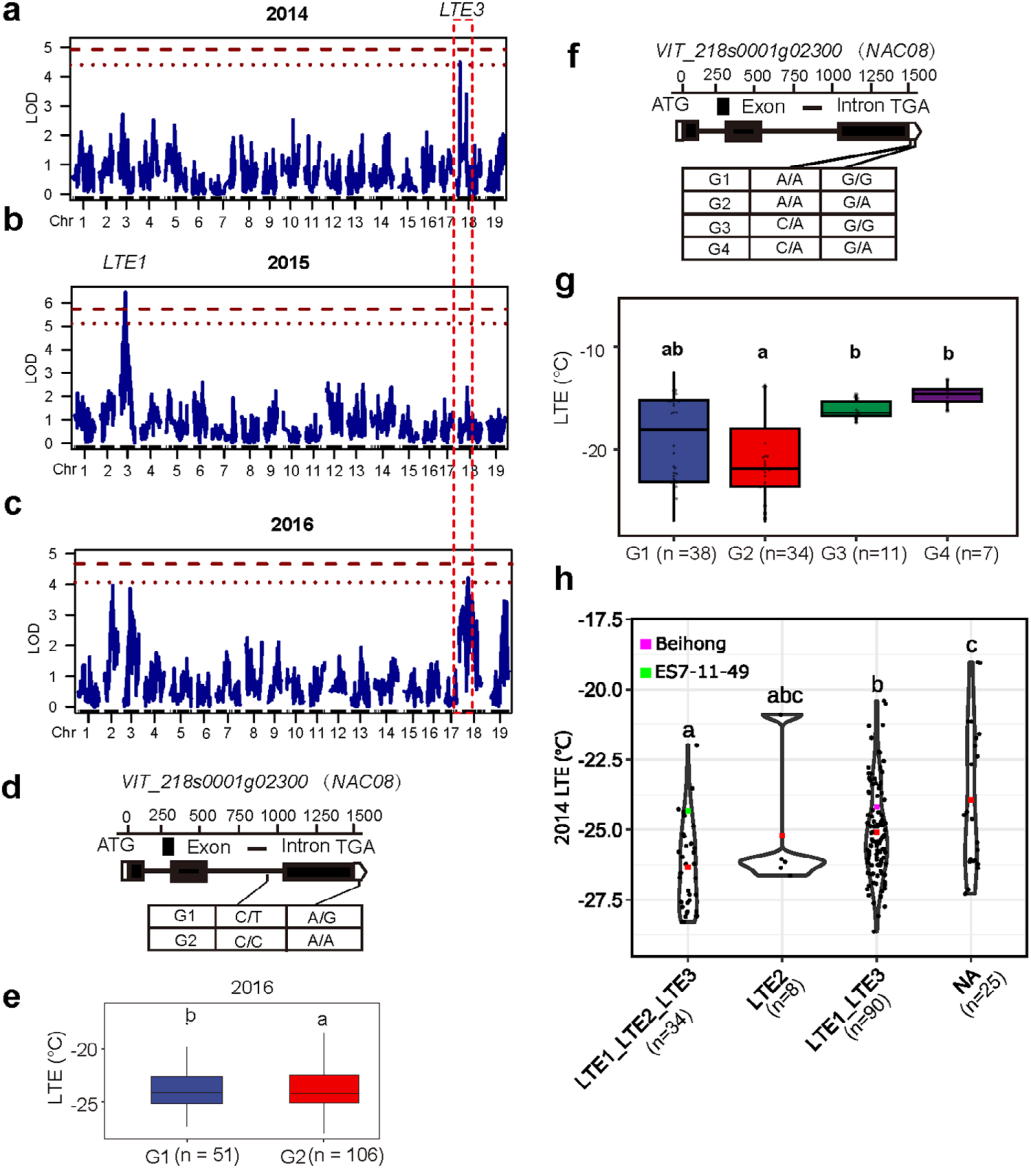
Genetic basis of cold tolerance. a–c) QTL mapping results for cold tolerance were obtained from the BH × ES population. Significant QTL mapping results were observed only in 2014, 2015, and 2016 among the analyzed years (2012–2016) (a–c). The red dotted line indicates the LOD threshold obtained from 1000 permutation tests, with a significance level of 0.05 (a–c). The red dashed line represents the LOD thresholds obtained from 1000 permutation tests, with a significance level of 0.01 (a–c). d) Structure of candidate gene *VIT_218s0001g02300* (*NAC08*) with its two major genotypes in the BH × ES population. e) Boxplots of LTE according to the two genotypes (G) of the candidate gene in the BH × ES population in 2016. f) Structure of candidate gene *VIT_218s0001g02300* (*NAC08*) with its four major genotypes in the germplasm resource population. g) Boxplots of LTE according to the four genotypes (G) of the candidate gene in the germplasm resource population in 2018. h) Dot plots illustrate the LTE variation according to different allelic combinations for *LTE1*, *LTE2* (Figure S19, Supporting Information) and *LTE3* in the BH × ES population. Progenies are depicted as black dots, and red dots indicate the medians of each category. The purple and green dots indicate the values for the parental alleles. NA indicates progeny carrying no favorable alleles. The presence of different letters above the boxplots in e,g,h indicates statistically significant differences (*p* < 0.05) in the distribution of values among different genotypes, as determined through the application of Tukey's test. The sample size was shown under each boxplot in e,g,h.

Low‐temperature exothermic analysis is a reliable way to determine the lethal temperature for grapevine winter buds,^[^ ^38^ ^]^ used here as an indicator of cold tolerance (LTE). In the BH × ES F_1_ population, LTE values ranged from −31.46 °C to −11.22 °C between 2012–2016, and from −26.99 °C to −11.33 °C in 2018 in the germplasm resource population (Figure S18, Table S17, Supporting Information). We conducted QTL mapping and GWAS on LTE data after Box‐Cox transformation (**Figures** **6**a–c; Figure S19, Supporting Information). The results of the analysis pointed to two novel QTLs (*LTE2* and *LTE3*) and one known QTL (*LTE1*)^[^ ^39^ ^]^ previously associated with cold tolerance (Table S18, Supporting Information). *LTE3* on chromosome 18 was detected by QTL mapping in the BH × ES F_1_ population over two growing seasons and by GWAS in the germplasm resource population (Figure 6; Figure S19, Supporting Information). This stable QTL explained 4.90%–7.77% of the variation in the LTEs (Table S18, Supporting Information). Candidate gene exploration showed that 244 genes were located in the *LTE3* confident intervals identified from the BH × ES F_1_ population (Table S19, Supporting Information). By contrast, only one gene (*NAC08*, *VIT_218s0001g02300*) was identified in the GWAS of the germplasm resource population (Figure S19f, Supporting Information). Notably, this *NAC08* transcriptional factor was listed among the 244 genes (Table S19, Supporting Information). Knowing that *NAC08* has been previously reported to enhance drought tolerance in transgenic *Arabidopsis*,^[^ ^40^ ^]^ it was chosen as a pertinent candidate gene for further validation.

Haplotypes of *NAC08* were analyzed (Figure 6d–g). In the BH × ES F_1_ population, two genotypes were identified that were associated with LTE values (Figure 6d,e), while in the germplasm resource population, four genotypes were related to LTE values (Figure 6f,g). Based on this, it was inferred that the gene *NAC08* plays a role in controlling cold tolerance. To assess the pyramiding impact of lower LTE alleles for cold tolerance, we examined the effects of *LTE1*, *LTE2*, and *LTE3 (NAC08)*. As anticipated, progeny carrying favorable alleles at *LTE1*, *LTE2*, and *LTE3* all exhibited lower levels of LTE, indicating a higher capacity for cold tolerance compared to those carrying only the *LTE1* and *LTE3* allele or none of the cold tolerance alleles (Figure 6h).

**Figure 7:**
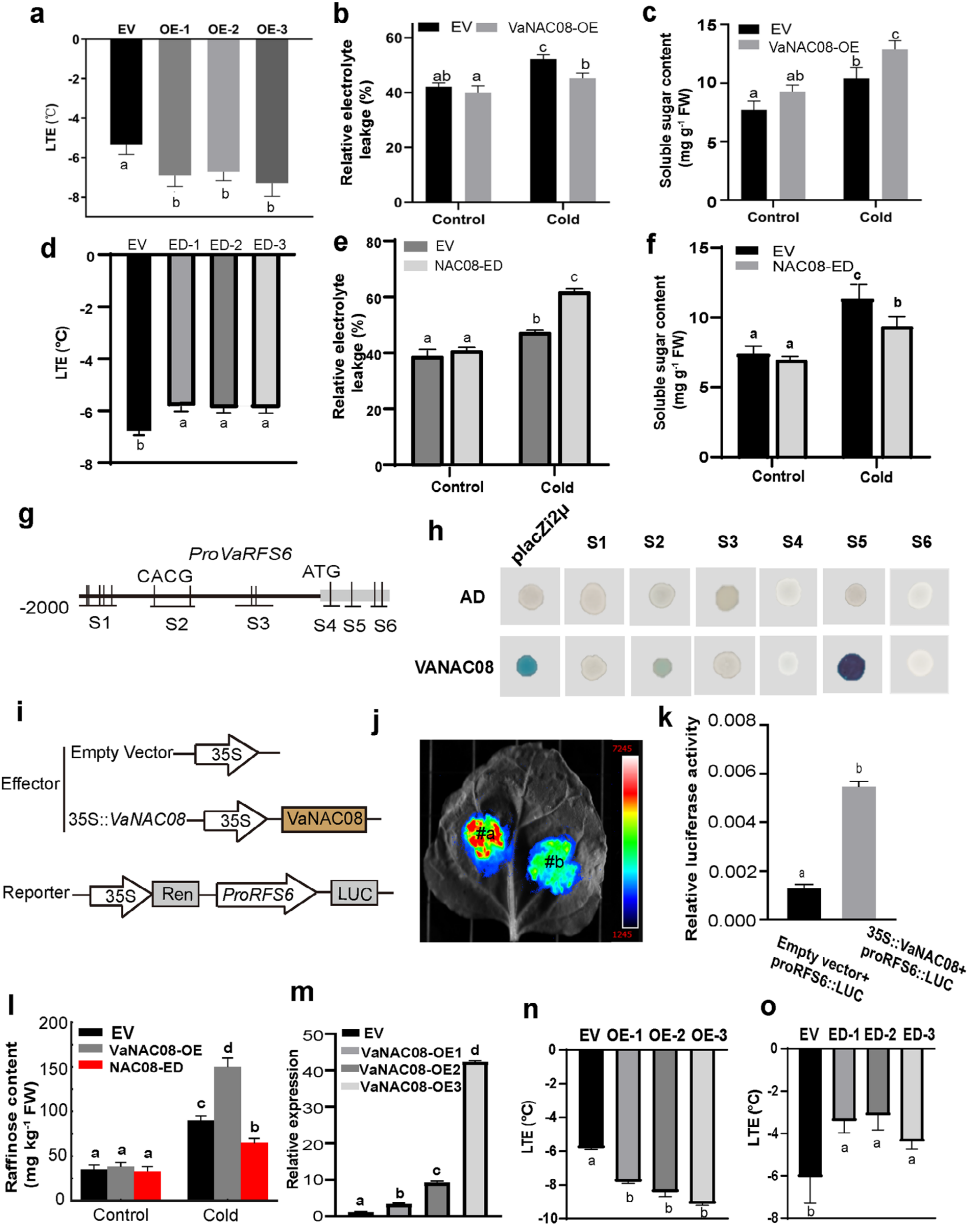
Overexpression of *VaNAC08* upregulates *VaRFS6* expression and enhances raffinose accumulation during cold stress. a, The LTE values (°C) of empty vector (EV) and overexpression (OE) transgenic lines of grape calli (OE‐1, OE‐2, and OE‐3). b) Electrolyte leakage in EV and OE lines of grape calli at room temperature (Control) and 4 °C for 4 h (Cold). c) Soluble sugar content (mg g^−1^ FW) in EV and OE lines of grape calli at room temperature (Control) and under cold stress (4 °C 4h). d) LTE value (°C) in EV and transgenic *NAC08* knockout (*NAC08‐ED*) of grape calli. e) Electrolyte leakage in *NAC08* knockout lines of grape calli at 4 °C for 4 hours. f) Soluble sugar content (mg g^−1^ FW) in *NAC08* knockout lines of grape calli at room temperature (Control) and under cold stress (4 °C for 4 h). g, A diagram of the *VaRFS6* promoter with VaNAC08 binding site (S1, S2, S3, S4, S5, CACG). h) Identification of VaNAC08 binding to the promoter of *VaRFS6* was achieved through yeast one‐hybrid assays. The *VaRFS6* promoter was constructed by fusing five fragments with the placZi2µ vector. The coding sequence of *VaNAC08* was combined with the activation domain of the pB42AD vector to produce NAC08‐pB42AD. Co‐transformation of proRFS6‐placZi2µ (S1, S2, S3, S4, S5) and NAC08‐pB42AD into yeast strain EGY48 resulted in yeast transformants that were able to grown on SD/‐Trp/‐Ura/Gal/Raf/X‐gal medium. Negative controls included the empty pB42AD vector (AD) and transformants containing AD and proRFS6‐placZi2µ (S1, S2, S3, S4, S5). i–k) VaNAC08 was identified to activate the expression of *VaRFS6* through dual luciferase gene reporter assays. The coding sequence of VaNAC08 was fused with the CaMV35S promoter from pCAMBIA‐2300 to serve as the effector. The promoter of *VaRFS6* was inserted into the pGreenII‐0800‐LUC vector as the reporter i) (#a). Both effectors and reporters were transformed into tobacco leaves, with the EV + *proVaRFS6::LUC* (#b) as the control j). The ratio of LUC/REN indicates the relative luciferase activity k). l) The raffinose content was quantified in the overexpression (OE) transgenic lines and *NAC08* knockout lines of grape calli under both room temperature (Control) and cold stress conditions (4°C for 4 hours). m) The expression of *RFS6* in *VaNAC08* overexpression transgenic lines. n,o) The LTE values (°C) in *VaRFS6* overexpression (*OE‐VaRFS6*) n) and knockout (*RFS6‐ED*) lines of grape calli o). Different letters (a, b, c) above the plots indicate that the values of the allelic group are significantly different (*p* < 0.05), as determined by Tukey's test. The sample size for each treatment is 3.

To validate the function of *NAC08* in cold tolerance, we first investigated the expression of *NAC08* in response to cold stress in both *V. amurensis* (the paternal parent of BH with strong cold resistance, Figure S2b, Supporting Information) and *V. vinifera* cv. Muscat Hamburg (the maternal parent of BH with weak cold resistance, Figure S2b, Supporting Information). The results showed that *NAC08* was upregulated by cold stress in both grapevine species, but more so in *V. amurensis* (Figure S20, Supporting Information). Sequence analysis revealed one SNP in coding sequences between *VaNAC08* and *VvNAC08* (Figures S21 and S22, Supporting Information). For functional analysis of *VaNAC08*, we developed *VaNAC08*‐overexpressing (VaNAC08‐OE) and *NAC08* knockout (NAC08‐ED) transgenic lines in ‘41B’ grape calli^[^ ^41^ ^]^ (Figures S23 and S24, Supporting Information). The cold tolerance of transgenic calli was evaluated by determining several key physiological parameters, including LTEs, relative electrolyte leakage, and soluble sugar content (**Figure** **7**a–f). Compared to the control lines transformed with empty vector (EV), VaNAC08‐OE lines had lower LTEs (Figure 7a), while the LTEs of NAC08‐ED lines were much higher (Figure 7d). Furthermore, after cold (4 °C) treatment, the VaNAC08‐OE lines had less electrolyte leakage and higher soluble sugar content, whereas the NAC08‐ED lines leaked more electrolyte and contained less soluble sugar (Figure 7b,c,e,f). As additional corroboration in whole plants, *Arabidopsis* plants overexpressing *VaNAC08* were generated, and 4‐week‐old transgenic plants alongside wild‐type (WT) plants were subject to cold and freezing treatment. VaNAC08‐OE plants had a higher survival rate compared to the WT plants after exposure to −8 °C for 4 h (Figure S25a,b, Supporting Information). Moreover, the electrolyte leakage from VaNAC08‐OE plants was significantly lower than from WT plants when treated at 4 °C for 4 d (Figure S25c, Supporting Information). These results indicate that VaNAC08 positively regulates cold tolerance in grape calli and *Arabidopsis*.

To discover how NAC08 regulates cold tolerance, transcriptomic data from ‘Muscat Hamburg’ under 4 °C cold stress was analyzed for gene co‐expression^[^ ^42^ ^]^ (Table S20, Supporting Information) and considered alongside RNA‐seq data obtained from VaNAC08‐OE and control EV calli under normal conditions. By combining the two datasets, as many as 399 were found to be both tightly co‐expressed with *NACO08* (r > 0.9, p < 0.05, Table S21, Supporting Information) and up‐regulated by *NAC08* overexpression (VaNAC08‐OE). GO enrichment analysis of these genes highlighted 85 candidate genes annotated with the ‘response to stimulus’ term (Tables S22 and S23, Supporting Information). Cold tolerance has previously been associated not only with transcriptional regulators but also with phytohormones and important metabolites, including raffinose.^[^ ^43^ ^]^ Among the 85 cold‐tolerance gene candidates are two genes (*VIT_211s0016g05770* and *VIT_214s0066g00810*) known to be involved in the biosynthesis of raffinose, a sugar which has been reported to contribute to cold tolerance in grapevine.^[^ ^44^ ^]^ The expression of *VIT_214s0066g00810* in response to cold stress was very low. However, the gene *VIT_211s0016g05770* (named *RFS6* hereafter) was more highly expressed, so was further explored.^[^ ^43^ ^]^ Putative CACG elements predicted for NAC transcription factor binding were identified in the promoter and the first exon of *VaRFS6*, which was further divided into 6 fragments (S1‐S6) for subsequent DNA binding tests (Figure 7g). Yeast one‐hybrid assays demonstrated that VaNAC08 directly binds to the S2 and S5 regions of the *RFS6* promoter (Figure 7g,h). Dual‐luciferase reporter assays provided additional evidence that NAC08 can activate the expression of *RFS6* in vivo in tobacco leaves (Figure 7i–k). When overexpressed in grape calli, *VaNAC08* increased the raffinose content, while knocking out *NAC08* in grape calli lowered the content of raffinose (Figure 7l) under cold stress. It was noted that the expression of *RFS6* was upregulated in the VaNAC08‐OE lines (Figure 7m; Figure S26, Supporting Information). The role of VaRFS6 in cold tolerance was also assessed in grape calli overexpressing and knockout lines. VaRFS6‐OE had significantly fewer LTEs (Figure 7n), while the RFS6‐ED lines had more LTEs than the EV control lines (Figure 7o; Figure S27, Supporting Information). These results suggest that VaNAC08 may regulate cold tolerance by modulating raffinose accumulation through activating *VaRFS6* expression (Figure S28, Supporting Information).

### 2.8 Association Mapping of Flower Sex and Fine‐Mapping of the Sex‐Determining Region

**Figure 8:**
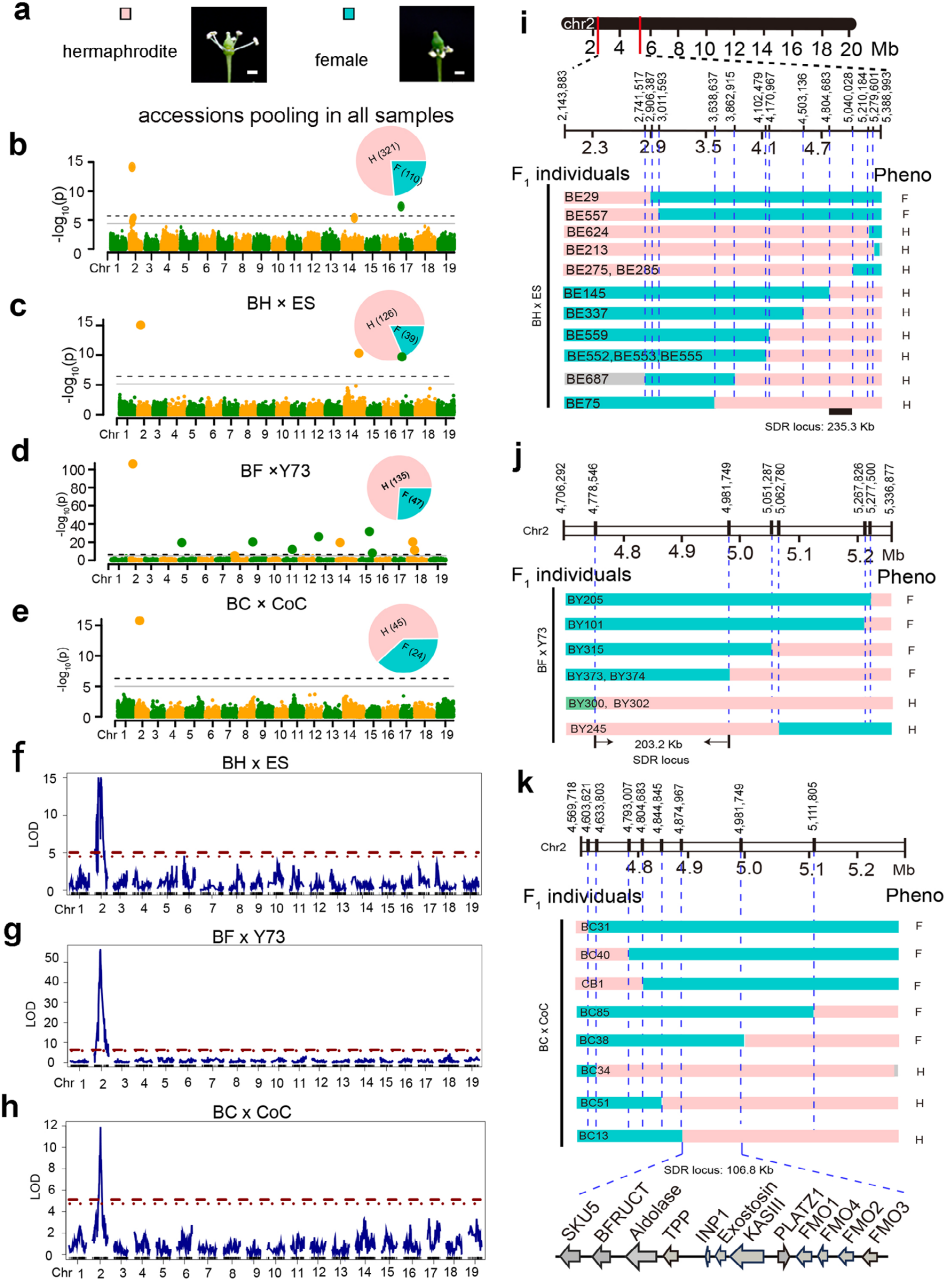
Association analysis of grapevine flower sex and subsequent fine‐mapping of the sex determining region (SDR). a, Representative photographs of flowers are shown with a scale bar of 1.0 mm. b–e) Manhattan plots display the GWAS results for flower sex in 454 *Vitis* pooled genotypes of the three breeding populations b), or separately genotypes of the F_1_ populations of BH × ES c), BF × Y73 d), and BC × CoC e). The GWAS analysis was performed using BLINK. Pie charts show the number of females (blue sector) and hermaphrodites (pink sector) in each sample. The grey solid lines show the significant threshold while the black dashed line shows the highly significant threshold of *p* values. f–h) QTL mapping results for flower sex are presented for the three breeding populations BH × ES (f), BF × Y73 (g), and BC × CoC (h). The red dotted line indicates the LOD threshold derived from 1000 permutation tests, with a significance level of 0.05. The solid red dashed line represents the LOD thresholds obtained from 1000 randomization trials, with a significance level of 0.01. i–k) Crossover mapping schemes are illustrated for recombination around the SDR locus in BH × ES (i), BF × Y73 (j), and BC × CoC (k) are shown. Names of individuals are given on the left and the phenotypes on the right. Each individual recombinant chromosome is depicted in pink for SDR hermaphrodite, blue for SDR female, and grey for underdetermined haplotypes. Additionally, the study also includes a comprehensive analysis of the markers examined in each F_1_ mapping population, the detailed region of the SDR that was mapped, and the genes situated within that region based on their relative distances in the PN40024 12X.v0 reference genome and 12X V2 gene annotations.

Cultivated grapevine varieties tend to have hermaphrodite flowers but single‐sex flowers are characteristic of dioecious wild *Vitis* species (**Figure** **8**a). The 3:1 ratio of individuals with hermaphrodite flowers to individuals with female flowers is characteristic of discrete variation in the three populations (Figure S29a, Table S24, Supporting Information), indicating it as a trait under monogenic control as reported previously.^[^ ^45^ ^]^ Association mapping was performed to investigate the genetic determinants of flower sex using the grapevine 200K SNP array (Figure 8b–h). The GWAS analysis was conducted four times. The initial analysis entailed pooling the F_1_ individuals of all three populations (BF × Y73, BH × ES, and BC × CoC) along with the 6 parents for GWAS (Figure 8b; Table S25a, Supporting Information). The remaining three analyses were carried out separately for each population, employing all F_1_ individuals for GWAS (Figure 8c–e; Table S25a, Supporting Information). Each analysis utilized a specific panel of SNP markers, a total of 152,782, 74,496, 135,600, and 100,460 markers, respectively, for the four rounds (Figure 8b–e). The GWAS detected two QTLs, one known QTL on chromosome 2^[^ ^46^, ^47^, ^48^, ^49^ ^]^ and a novel QTL on chromosome 5. QTL mapping was also conducted for the three populations (Figure 8f–h). The combined results of GWAS and QTL mapping identified the previously known QTL as a consistent main‐effect QTL.^[^ ^46^, ^47^, ^48^, ^49^ ^]^ This QTL was detected across all four GWAS datasets and specifically in the three F_1_ populations during QTL mapping, suggesting its consistent presence and significance in determining flower sex (Figure 8b–h; Table S25a,b, Supporting Information).

To more finely map the location of the mutations responsible for the sex‐determining region (*SDR*) QTL, a genetic strategy was employed. F_1_ individuals that inherited either the mutant haplotype (*sdr*) for the female allele or the hermaphrodite allele from the progenitor were observed. This confirmed that the female factor is recessive. The F_1_ population was then screened for meiotic recombinants based on marker and phenotype observations. A total of 14 recombinants were identified in the BH × ES population, 2 females and 12 hermaphrodites. The interval between recombination points spanned a physical distance of 235.3 kb. To further delimit the location of the *sdr* mutations distinguishing the female and the hermaphrodite alleles of the *SDR* locus, additional recombinants were sought in BF × Y73 and BC × CoC progeny. In the BF × Y73 progeny, eight recombinant F_1_ individuals were identified. Crossover mapping through genotyping helped delimit the *sdr* mutations to a physical interval of 203.2 kb. Similarly, in the BC × CoC progeny, eight recombinant F_1_ individuals were found, and the *sdr* mutations were delimited to a physical interval of 106.8 kb. This fine‐mapped *SDR* locus interval encompasses 12 genes that are candidates for the QTL. Although these 12 genes are located within the SDR region, some differences were noted compared to previous reports.^[^ ^48^, ^49^ ^]^ We believe this discrepancy is because the male haplotype is not present in our populations, so the male flower phenotype is not segregating. Consequently, our fine‐mapping focuses on distinguishing between the hermaphrodite and female alleles.

### 2.9 Genomic Loci Linking to Multiple Traits

We investigated potential pleiotropic loci where multiple traits co‐localized (Figures S30 and S31, Supporting Information). We categorized the recorded phenotypic traits into three distinct classes: berry‐related traits (including color, shape, soluble sugar, and organic acid), cold resistance, and flower sex. We identified a total of two genomic loci linking to multiple types of traits, located on chromosomes 6 and 17, respectively. (Figure S31, Table S26, Supporting Information). The most prominent locus at chromosome 6 within the region from 5.7 to 7.5 Mb was co‐localized with the highest number of traits for sugar and acids by both GWAS and QTL mapping. The other locus at chromosome 17 within the region from 3.8 to 4.4 Mb was co‐localized with color and shape traits measured from HTP by GWAS. It is important to note that the physical overlap of loci associated with traits does not automatically imply the same SNPs are causally related to those traits.

## 3 Discussion

High‐density genetic markers are a prerequisite for analyzing complex traits in grapevine. The high‐density 200K Axiom grape array is a significant step up from previous marker sets.^[^ ^15^, ^21^ ^]^ The advantages of the new array have been demonstrated in constructing a highly concentrated genetic map (Figure 3e) and elucidating pertinent marker trait associations for various grapevine traits. The expansive genome‐wide coverage and substantial number of SNP markers ensure the array can be effectively employed to finely map genetic loci associated with numerous specific valuable traits. This is particularly important because the intricate genetic architecture underlying these traits needs to be understood to enable targeted breeding efforts. The inclusion of a diverse array of genotypes, spanning Eurasian accessions, accessions from Northern America and East Asia, HYB, and a wild relative, *Ampelopsis brevipedunculata*, enhances applicability across a broad spectrum of genetic backgrounds (Figure 1b; Table S1, Supporting Information). The exceptionally close agreement between the physical and genetic positions of the SNP markers indicates that the *Vitis* genetic map constructed using the 200K Axiom platform is robust (Figure 3f). Overall, these are valuable resources for grape genetic research, genomic selection, and breeding programs. The amalgamation of the extensive SNP dataset, high‐density SNP array, and diverse set of genotypes establishes a strong foundation for exploring the genetics of economically valuable traits and implementing marker‐assisted breeding strategies to enhance grape cultivation.

We successfully integrated phenome and genome data by harnessing the potential of the HTP platform in conjunction with the 200K Axiom SNP array (Figure 1c). The HTP platform was cost‐effective and efficient in accurately capturing critical berry shape and color traits. Compared to previous studies,^[^ ^50^ ^]^ we used the berry pedicel as a visual anchor from which to determine fruit orientation. We noted and visualized which steps were error‐prone during processing by providing real‐time sequential visuals of berry handling procedures. These strategic enhancements significantly increased the accuracy and reliability of the measurements. Phenotypic features were comprehensively described through multiple statistical indicators and analyzed by PCA (Figure 1c). This allowed an unbiased and global approach to understanding the distributions of traits. Furthermore, the integration of GWAS and QTL mapping of shape and color features allowed us to corroborate the pertinence of established genes and identify novel hotspots influencing berry morphology and coloration. This enriches our comprehension of the genetic regulation of these pivotal traits.

We have pinpointed five high‐priority candidate genes particularly linked to crucial grapevine attributes based on haplotype analysis and gene annotation. *VIT_204s0023g01930* encodes a bHLH transcriptional factor (*bHLH017*) which is tightly linked with the berry shape index as assessed by HTP over three years (Figure 4h–j). *VIT_213s0019g00740* encodes an ACT domain repeat protein and may act as a suppressor in glucose signaling pathways,^[^ ^35^ ^]^ and therefore is a promising target for enhancing sugar levels in grapevine cultivars (Figure S13i,j, Supporting Information). We identified two known genes, *ALMT1* and *FUSC2* ^[^ ^37^ ^]^ (Figure 5e,f), as influencing organic acid levels. Additionally, the gene *VIT_218s0001g02300* (*NAC08*), located within the *LTE3* region, encodes a NAC transcription factor that has been verified to positively regulate cold tolerance (Figures 6 and 7) by activating the expression of *VaRFS6* and increasing raffinose content (Figure 7; Figure S28, Supporting Information). These candidate genes hold significant potential for indirect breeding approaches, such as marker‐assisted selection (MAS), as well as for genetic engineering techniques, including CRISPR‐based genome editing and cloning of functional genes. Notably, the validated cold‐tolerance gene *NAC08* contributes to enhancing grapevine cold tolerance so could be a prospective target for the development of improved grapevine varieties. Multiple genomic loci show significant correlations with different traits, and haplotype analysis has revealed two loci with notable phenotypic variation. These are located on chromosome 6, associated with sugar and acid content, and chromosome 17, associated with color and shape (Figures S30 and S31, Table S26, Supporting Information).

In summary, the assembly and use of a 200K Axiom SNP array and high‐throughput phenotyping tools have revealed more detail about the genetic regulation of berry quality, cold tolerance, and floral development in grapes. The joint development of the SNP array using the latest re‐sequencing data from grape cultivars, and the accurate measurement of berry features vastly enhances the precision and efficiency of procedures for breeding. The 200K Axiom SNP array results were amenable to GWAS and QTL mapping, and, in combination with genetic techniques, to narrowing down the QTL region to determine the exact location of the mutation causing the trait. This strategy can be used to identify genetic variants that influence specific traits. Understanding the additive effects of these mutations is crucial for breeders when deciding preferences for berry quality traits. Although the grapevine was domesticated ≈11 000 years ago, it still relies on breeders and agricultural scientists to develop new varieties to this day. The implementation of high‐density SNP arrays and HTP tools will greatly accelerate the precise identification of more genes and improve the quality and sustainability of grapevine cultivars. Collectively, HTP tools and the 200K SNP array highlight their potential in shaping the genetic architecture of complex traits in grapevine.

## 4 Experimental Section

### Plant Genomic and Genetic Resources

A collection of re‐sequenced genome sequences consisting of 313 *Vitis* genotypes and one *Ampelopsis brevipedunculata* genotype was sourced from Liang et al.^[^ ^28^ ^]^ (Table S1, Supporting Information). High‐quality reads (Phred quality score ≥ 25) that did not contain adapters or poly‐N sequences were selected using NGSQCToolkit_v2.3.3^[^ ^51^ ^]^ for the design of the Affymetrix 200K Axiom SNP array.

To assess the genotyping accuracy of the 200K SNP array, fresh leaves from 471 samples were collected from the grape genetic resource vineyard of the Institute of Botany (Chinese Academy of Sciences, Beijing, China). The grapevines were cultivated and managed following local viticulture standards, and genomic DNA was isolated using the CTAB protocol.^[^ ^52^ ^]^ The 200K Axiom genotyping array was used to analyze three F_1_ mapping populations along with their respective parents, which contained in total 471 accessions (Table S5, Supporting Information). The inter‐species F_1_ progeny included 187 individuals from the BF (*V. bryoniifolia* × *V. vinifera*) × Y73 (*V. vinifera*) population, 196 individuals from the BH (*V. vinifera* × *V. amurensis*) × ES ((*V. labrusa* × *V. riparia*) × *V. vinifera*) population, and 82 individuals from the BC (*V. vinifera* × *V. amurensis*) × CoC (*V. labrusca*) population (Figure S2, Supporting Information). The integrity of the extracted DNA was assessed by agarose gel electrophoresis, while the concentration was determined using a Nanodrop 2000 spectrophotometer (Thermo Fisher Scientific, USA).

All progenies from the BC × CoC, BF × Y73, and BH × ES populations were examined for kinship to their respective parents using a 200K Axiom SNP array. Individuals that exhibited a higher degree of genetic similarity with the female parent and a lower degree with the male parent compared to true progeny are more likely to have arisen from pollen contaminants or self‐hybridization (Figure S31, Supporting Information). One such individual in the BH × ES population and six in the BC × CoC population (Figure S32, Supporting Information) were excluded from further analysis to safeguard the accuracy and reliability of the genetic data.

To validate the high‐throughput grape berry phenotyping system (HTP), 14 grape cultivars were selected from the grape germplasm vineyard located at the Institute of Botany (Chinese Academy of Sciences, Beijing, China) (Figure S5, Supporting Information). In parallel, the F_1_ population of the BF (*V. bryoniifolia* × *V. vinifera*) × Y73 (*V. vinifera*) cross was phenotyped over three growing seasons (2021‐2023). Each individual within this population had 3–5 vines and bore fruit during the experiment. The F_1_ population was phenotyped at maturity (BBCH 89) in each season, with 100 berries collected from three vines representative of each genotype. The F_1_ population served as the basis for phenotypic and association analysis using the phenotyping data.

To assess cold tolerance in a population of germplasm resources, 101 accessions from various regions were chosen and are currently planted at the Institute of Botany (Chinese Academy of Sciences, Beijing, China) (Table S17, Supporting Information).

### SNP Calling and Array Development

The present study employed the SNP‐calling pipeline previously described by Liang et al.^[^ ^28^ ^]^ The pipeline parameters produced 101.6 million high‐quality SNPs from the whole‐genome re‐sequencing analysis of 313 genotypes along with one genotype of *Ampelopsis* that was distributed across 19 chromosomes and some unanchored scaffolds. These SNPs were stored in the standard variant calling format (VCF) and the information was used for designing the 200K Axiom SNP array. To ensure the quality of the SNPs, the following filters were applied: 1) single‐copy locus filtering, where BLAST alignment was performed with 35 bp flanking up or downstream to avoid multi‐alignment errors, 2) missing rate filtering of ≤0.3, minor allele frequency filtering of ≥ 0.05, and sequencing depth filtering of ≥4, and 3) screening for interference from up or downstream SNPs by discarding any SNP located within a distance of less than 35 bp from the target SNP. Thus, a total of 204 860 candidate SNPs were selected for the preliminary design of a 200K Axiom SNP array. The SNPs were then classified into four categories (‘recommended’, ‘neutral’, ‘non‐recommended’, and ‘not‐possible’) using proprietary software. Ultimately, the 200K Axiom grapevine genotyping array was comprised of 174,464 unique SNP markers.

### Validation of the 200K Axiom SNP Array

Genomic DNA was hybridized to the 200K SNP array by China Golden Marker Biotech Co., Ltd. (Beijing, China, https://www.goldgene.com), followed by genotyping using the GeneTian platform. The resulting CEL files underwent quality control and genotype calling. SNP quality control was performed using the Affymetrix Power Tools (APT) software packages, which measured the development quality check (DQC) value based on the signal‐to‐noise ratio of non‐polymorphic probes. DQC values were used to assess acceptable resolution, but sample call rate was also considered. The genotyping data were analyzed using diploid threshold configurations in the Axiom Analysis Suite v4.0.3 (Thermo Fisher Scientific, Applied Biosystems, USA). SNPs were categorized into six main groups, including ‘Poly High Resolution’, ‘No Minor Hom’, ‘Off‐Target Variants’, ‘Mono High Resolution,’ ‘Call Rate Below Threshold,’ and ‘Other’ based on genotyping data quality (Figure S3, Supporting Information).

### Population Genetics Analysis

Statistical analyses were performed exclusively on high‐quality SNPs (174,464) with minor allele frequency ≥10% and missing rates <10%. SNPs in linkage disequilibrium were further filtered using PLINK (v1.90b6.24)^[^ ^53^ ^]^ employing a window size of 50 SNPs and a R^2^ threshold of 0.2. The maximum‐likelihood tree was constructed using SNPphylo^[^ ^54^ ^]^ with 1,000 bootstrap replications and visualized using the iTOL tool (https://itol.embl.de).^[^ ^55^ ^]^ PCA was conducted using PLINK (v1.90b6.24),^[^ ^53^ ^]^ and the results were visualized using the ggplot2 package in R software.^[^ ^56^ ^]^ The population structure was analyzed using the ADMIXTURE (v1.3)^[^ ^32^ ^]^ program with the number of genetic clusters *K* varied from 2 to 5, and the bar plot function in R packages^[^ ^56^ ^]^ was used to visualize the results.

### Pipeline for HTP

HTP pipelines conventionally encompass four distinct methodical stages. The primary objective of the pipeline was to conduct a rapid and precise assessment of a wide array of phenotypic traits across a substantial quantity of grape berries. The platform includes a camera, a photography booth, and a custom plastic board, which provides a cost‐effective and portable facility for acquiring many grape berry images rapidly. First, for sample preparation and collection, 100 berries were selected from a minimum of 3 ripe clusters for each genotype. The berries were carefully separated from the clusters using scissors, preserving a 2–5 mm pedicel for anchoring the berry orientation during subsequent image processing. All berries were neatly arranged on the custom plastic board, providing a grid of 10 × 10 depressions to process a maximum of 100 berries at once and to easily keep the berries in position (Figure 1c). Subsequently, high‐resolution imaging systems (≥ 200 pixels per inch), including specialized scanners or cameras, were employed for the acquisition of berry images. To standardize image acquisition, rigorous attention was paid to maintaining consistent lighting conditions and ensuring that the berries were positioned parallel to the pedicel and the plane of the plastic board (Figure 1c). Third, during image processing and segmentation, acquired images were corrected for color and perspective as necessary to rectify any disparities. Subsequent segmentation of the images distinguished the berries from other elements. A crucial step involved utilizing the pedicel position as an anchor point to effectively isolate images of individual grape berries, thus minimizing background noise. More importantly, the pedicel as an anchor is to have a fixed initial point to make the extracted feature more reliable. Last, for trait extraction, calculations were performed by determining the ratio with respect to the scale of the custom plastic board, facilitating the computation of the pixel‐to‐real size relationship. Phenotypic traits were then extracted through a comprehensive assessment of pixel count, distances, color space values within pixels, and subsequent feature calculations based on this information. Briefly, the automated phenotypic extraction algorithms were composed of four parts: perspective correction relative to the custom plastic board, color correction based on the standard color card, image semantic segmentation based on berry position recognition, trait extraction, and visualization. The results obtained were recorded in csv format for in‐depth analysis.

### GWAS and QTL Mapping

Bayesian‐information and Linkage‐disequilibrium Iteratively Nested Keyway (BLINK),^[^ ^57^ ^]^ fixed and random model circulating probability unification (FarmCPU),^[^ ^58^ ^]^ and mixed linear model (MLM) were employed^[^ ^59^ ^]^ (Extended View Figure 3), along with the general linear model (GLM) in TASSEL 5.0.^[^ ^60^ ^]^ BLINK^[^ ^57^ ^]^ model was selected for presentation in the main figures. The effective number of uncorrelated SNPs (N) was calculated using the Genetic Type I Error Calculator (v.0.2).^[^ ^61^ ^]^ To effectively control the genome‐wide type 1 error rate, we set *p*‐value thresholds for significance (1/N), applied false discovery rate (FDR) adjustment^[^ ^62^ ^]^ and high significance (0.05/N). For the entire population, the *p*‐value thresholds were 1.92 × 10^−5^ for significant and 9.58 × 10^−7^ for highly significant. For the BH (*V. vinifera* × *V. amurensis*) × ES ((*V. labrusca* × *V. riparia*) × *V. vinifera*) population, the *p*‐value thresholds were 4.00 × 10^−5^ for significant and 2.00 × 10^−6^ for highly significant, respectively. For the BF (*V. bryoniifolia* × *V. vinifera*) and Y73 population, the *p*‐value thresholds were 5.98 × 10^−5^ for significant and 2.99 × 10^−6^ for highly significant. For the BC (*V. vinifera* × *V. amurensis*) × CoC (*V. labrusca*), the *p*‐value thresholds were 5.37 × 10^−5^ for significant and 2.69 × 10^−6^ for highly significant. Finally, for the 101 accessions, the *p*‐value thresholds were 3.21 × 10^−6^ for highly significant.

QTL detection for each trait was conducted by generating a four‐way cross object using the R package qtl,^[^ ^63^ ^]^ followed by a 1D one‐QTL genome scan employing the Haley–Knott regression method. The stepwise QTL function employed a model selection approach to accurately identify genuine QTLs effectively while minimizing the inclusion of unrelated loci.^[^ ^64^ ^]^ QTL mapping was carried out utilizing a univariate approach, analyzing one trait at a time. To expedite the computation, we parallelized the permutations into 1,000 tasks distributed across a high‐performance server (Dell Precision 2580, Dell Inc., USA) using a customized R script. Subsequently, the results of these permutations were automatically collected, and distinct penalties for each trait/year combination were computed. These penalties were then utilized for the selection of significant QTLs at a significant level of α = 0.05.

### Quantitative Analyses of Anthocyanins, Sugars, and Acids

A total of 187 genotypes from the BF (*V. bryoniifolia* × *V. vinifera*) × Y73 (*V. vinifera*) population were sampled at maturity. Each genotype was subjected to three replicates, with one or two berry clusters harvested from each replicate. The estimation of the maturity date was determined using the method described by Chen et al.^[^ ^65^ ^]^ The berry skin was carefully separated from the berries and stored at −80 °C. HPLC analysis was conducted to assess the anthocyanin content in the berry skin, following the method outlined by Pereira et al.^[^ ^66^ ^]^ A total of 196 genotypes from the BH (*V. vinifera* × *V. amurensis*) × ES ((*V. labrusca* × *V. riparia*) × *V. vinifera*) population were also sampled at maturity using the same sampling method. The measurement of soluble sugars and organic acids in the berries was conducted following the method described by Chen et al.^[^ ^65^ ^]^ The normality of the berry soluble sugar and organic acid content distributions was assessed using the Shapiro‐Wilks^[^ ^67^ ^]^ test, and non‐normal distributions were transformed using the Box‐Cox transformation for QTL mapping or GWAS.

### Cold tolerance Evaluation with Differential Thermal Analysis and Physiological Assays

The cold tolerance of dormant buds was assessed by differential thermal analysis (DTA) following protocols described by Mills et al.^[^ ^68^ ^]^ and Chai et al.^[^ ^69^ ^]^ The DTA system comprised a Tenney Environmental Test Chamber (model T2C, Thermal Product Solutions, USA) equipped with ten thermoelectric modules (TEMs) and linked to a programmable freezer and a Keithley Multimeter Data Acquisition System (DAS) (model 2700‐DAQ‐40). The program of the freezer consisted of a gradual decrease in temperature from 4 °C to −40 °C over 10 h, and then quickly returning to 4 °C within 45 min. The DAS captured signals from each TEM every 15 s. Exotherm reactions were manually detected from a graph showing the thermistor output on the *x*‐axis and the difference between the loaded‐TEM output and the empty‐TEM output on the y‐axis. The low‐temperature exotherms (LTE) were identified as a dependable indicator for cold tolerance.^[^ ^69^ ^]^

The LTE of each individual in BH × ES progeny was measured over six growing seasons (2012, 2013, 2014, 2015, and 2016) and the LTE of the 101‐germplasm collection was also assessed in 2018. Primary shoots were collected in late October of each year and dormant buds from the 3rd to 8th nodes were used for LTE determination. Buds flanked by ≈0.5‐1 mm of cane tissue were excised with a smooth cutting surface, and then temporarily stored on moist absorbent paper in a petri dish. Six buds were placed on each TEM for the LTE measurement, and data from three TEMs of the same genotype were considered as three biological replicates. The LTE distributions were normal for 2013 and 2016, but nonnormal for 2012, 2014, and 2015 according to the Shapiro–Wilk test.^[^ ^67^ ^]^ The values of non‐normal distributions were transformed using the Box‐Cox transformation for QTL mapping or GWAS.

The electrolyte leakage of *Arabidopsis* and 41B cells was assessed following the protocol outlined by Li et al.^[^ ^70^ ^]^ This assay was performed in triplicate. The soluble sugar content of 41B cells was carried out by the same methods using isolation kits (Solarbio). Five independent biological replicates and three technical replicates were carried out.

### Gene Cloning

Genomic DNA was extracted from the leaves of both *V. amurensis* and ‘Muscat Hamburg’ utilizing the Super Plant Genomic DNA Extraction Kit sourced from Tiangen (China, Cat: DP360). Total RNA was isolated from the leaves of these two plant species and used for cDNA synthesis, adhering to the manufacturer's guidelines of the HiScript III 1st Strand cDNA synthesis Kit (Vazyme). The full‐length coding sequences (CDS) of *NAC08* and *RFS6* were selectively amplified from the cDNA libraries of *V. amurensis* and ‘Muscat Hamburg,’ respectively, through PCR using the KOD‐Plus‐Neo Kit from TOYOBO. These PCR products were then cloned into the pLB cloning vector, also supplied by Tiangen, before proceeding with Sanger sequencing analysis. This approach involved cDNA synthesis according to the manufacturer's instructions. The full‐length CDS of *NAC08* and *RFS6* were amplified from the prepared cDNA libraries of *V. amurensis* and ‘Muscat Hamburg,’ respectively, via PCR using the KOD‐Plus‐Neo Kit (TOYOBO). The amplified fragments were then cloned into the pLB cloning vector (TIANGEN) for Sanger sequencing assay.

### Gene Transformation, Targeted Mutagenesis, and Cold Treatments

The coding sequences of *VaNAC08* and *VaRFS6* were individually inserted into the overexpression vector pCAMBIA2300 driven by the promoter of the CaMV 35S. The transgenes were transformed into 41B calli and *Arabidopsis thalina* using *A*. *tumefaciens*‐mediated transformation. The presence and expression of the transgenes were confirmed by PCR and RT‐PCR. Three transgenic lines were selected for further analysis. Transgenic plants were screened on 1/2 MS medium with 50 mg L^−1^ kanamycin. T_3_ homozygous transgenic lines were used for freezing treatments. To implement the freezing procedure, surface‐sterilized seeds were germinated and grown on plates containing 1/2 MS medium for 4 weeks. The temperature was gradually decreased at a rate of 1 °C per minute until it reached −8°C, and the plants were kept at this temperature for 4 h. Then the plants were placed in a temperature‐controlled chamber at 4 °C for 12 h before being reintroduced to standard growth conditions for 3 days. The freezing treatment was carried out without illumination, and survival rates and electrolyte leakage were examined after the plants had recovered to evaluate the impact of the treatment.

To knock out *NAC08* and *RFS6* genes in grapes, different targets were designed to target the exon of *NAC08* and *RFS6* using the target design tool of CRISPR‐GE (http://skl.scau.edu.cn/targetdesign). The designed sgRNAs were selected for designing target sgRNAs based on their location in the gene and off‐target possibilities. The 20‐bp sgRNA sequence was ligated into pYLCRISPR/Cas9 vector. To detect targeted mutagenesis, genomic DNA was extracted from induced calli samples, followed by amplification of the DNA fragment containing the target sequence. The PCR amplicons were then cloned into the pLB vector, and subsequently, 20 clones from each callus were analyzed using Sanger sequencing.

### RNA Sequencing Analysis

RNA of VaNAC08‐OE and EV grape calli was extracted for sequencing. Sequences were trimmed to a minimum length of 90 bp using TRIMMOMATIC (v0.36)^[^ ^71^ ^]^ and aligned to the PN40024 12X.v2^[^ ^72^ ^]^ reference genome with TopHat (v2.1.1).^[^ ^73^ ^]^ Expression levels were quantified using CUFFLINKS (v2.1.1),^[^ ^74^ ^]^ and differential gene expression analysis was conducted using CUFFDIFF (v2.2.1)^[^ ^74^ ^]^ using criteria of FDR < 0.05 and |log_2_ FC| > 1. The RNA‐seq data from VaNAC08‐OE and EV calli were stored in the National Genomics Data Center (NGDC) database (PRJCA028782). The transcriptomes of ‘Muscat Hamburg’ after 0, 2, 4, 8, 24 h at 4 °C are from Wang et al.^[^ ^42^ ^]^

### Yeast One‐Hybrid Assays

The pB42AD vector was used to clone the coding sequence of *VaNAC08*, while the placZi2µ vector was employed to ligate the promoter sequences of *VaRFS6*. Subsequently, the NAC08‐pB42AD construct was co‐transformed with the proRFS6‐placZi2µ (S1, S2, S3, S4, S5, S6) construct into yeast strain EGY48. Following a 3‐day incubation on SD/‐Trp/‐Ura selection media, the transformants were transferred to SD/‐Trp/‐Ura/Galactose/Raffinose selection media supplemented with 80 mg L^−1^ X‐Gal for color development.

### Transient Luciferase (LUC) Expression Assay

The promoter sequence of *VaRFS6* was cloned into the pGreenII‐0800‐LUC vector to create the reporter construct *proVaRFS6::LUC*. The pCAMBIA2300‐VaNAC08 vector was utilized as the effector. Both the effector and reporter constructs were transformed into the *A. tumefaciens* strain EHA105 and co‐transformed into tobacco leaves. The *Agrobacteria* were cultured overnight and then resuspended in infiltration buffer (10 mM MgCl_2_ and 10 mM MES) to a final OD600 of ≈0.5. The LUC and REN activities of the infiltrated leaves were measured using the Dual‐Luciferase Reporter Assay System (E1910, Promega, USA). The control consisted of the empty vector (EV) co‐transformed with *proVaRFS6::LUC*.

### Quantification of Raffinose

Raffinose was extracted and quantified from grape calli using a method described by Dixit et al.,^[^ ^75^ ^]^ utilizing high‐performance liquid chromatography (HPLC).

### Statistical Analysis

The normality of each data distribution was assessed using the Shapiro–Wilk test,^[^ ^67^ ^]^ and the corresponding *p*‐values are reported. Different letters above the bars indicate significant differences among the groups (p < 0.05), as determined by Tukey's test.^[^ ^76^ ^]^ Error bars represent the standard deviation (SD) from triplicate technical repeats. All statistical analyses were performed using R.^[^ ^56^ ^]^

## Conflict of Interest

The authors declare no conflict of interest.

## Supporting information



Supporting Information

Supplemental Table 1

## Data Availability

The data that support the findings of this study are available in the supplementary material of this article.
