# Supplementary material for: Integrating Dense Genotyping with High‐Throughput Phenotyping Empowers the Genetic Dissection of Berry Quality and Resilience Traits in Grapevine
Source: Adv Sci (Weinh). 2025 May 8;12(29):2412587. doi: 10.1002/advs.202412587 (PMC12362751; doi:10.1002/advs.202412587)
Supplement: Supplementary file 1 — Supporting Information [file ADVS-12-2412587-s002.docx]

**Supporting Information**

**Figure S1.** Grapevine chromosome distributions of markers from **(a)** 2K rhAmpseq^15^, **(b)** 18K SNP array^21^, and **(c)** the current 200K Axiom^®^ SNP array.

**Figure S2.** The pedigree of the three F_1_ breeding populations BF × Y73 **(a)**, BH × ES **(b)**, and BC × CoC **(c)**.

**Figure S3. SNP calling classifications.** Six different categories of SNPs were identified by SNP calling with Axiom Analysis Suite v4.0.3 (Thermo Fisher Scientific, Applied Biosystems, USA). Symbols indicate individual SNPs. Different color ellipses are used to indicate different clusters. The different color points signify different SNP categories.

**Figure S4. Population structure analysis of 454 Vitis genotypes.** a, Population admixture of 454 Vitis genotypes. Each genotype is depicted as a vertical bar, with the length of each colored segment indicating the proportion of ancestral contributed by different populations. b, Cross-validation error estimation plot for the population structure, relating to Supplementary Figure 3b. K = 3 was considered a sensible modeling choice.

**Figure S5. Grape genotypes of different shapes and colors for validating the HTP tool.** Fourteen grape cultivars at maturity **(a)** and their observed features distribution **(b)** were contained. The 14 cultivars from left to right, top to bottom were Shanhou 14, Red Palestine, Hongze, Russia Concor, Rou Dingxiang, Bai Aolin, Ziyu, Niagara, Shanhou 16, Izu Kam, Concord, Black Rose, Shine-Muscat, Sweet-Sapphire. The observed features distribution **(a)** contains berry size (such as length, width, and projected area), shape features (such as shape index, eccentricity, and circularity) and color features (such as mean values in different color spaces, color strength, and color ratios) based on the CIE Lab color space (L: Lightness, a: Red/Green Value, b: Blue/Yellow Value).

**Figure S6. Correlation of shape and color features of different grape varieties.** Note: Shape features include length (a), width (b), and shape index which is length/width (c), and the CIE Lab color space was used for color features evaluation. Color features include “L” which is lightness (d), “a” which is the ratio of green to red (e), and “b” which is the ratio of blue to yellow (f). The black dashed line represents the 1:1 line, while the blue solid line indicates the linear regression line. The linear regression equation, coefficient of determination (R²), and P-value are labeled in the upper left corner of each panel. A Lab colorimeter (FRU WR-18, China) was used to obtain the color values acquired. The bad correlation for the lightness can be neglected, since it represents the different light scenarios/illuminations during the measurement. The observation is closely tied to the methodology that is employed for measurements by the colorimeter. As illustrated in (d), the range of L values observed (25-50) is noted to be significantly narrower compared to those extracted (15-70). The discrepancy stems from the differing conditions under which measurements are taken: L values observed are assessed at a close distance (< 1 cm) under intense lighting, whereas images utilized for extracting L values are captured from a greater distance (> 50 cm) in relatively dimmer lighting conditions. The variation range of lightness on the berry skin is compressed by strong illumination during close-distance measurement, resulting in an underestimation of the L value. A reduced correlation for lightness measurements is consequently produced by this methodological difference, underscoring the necessity for a careful interpretation of such data within the context of the measurement techniques that are employed.

**Figure S7. PCA classification based on different feature groups.** Note: Principal component analysis (PCA) was used for dimension reduction (PC1 & PC2) and classification of different cultivars based on shape features **(a)**, color features **(b)**, all features **(c)**, and different genotypes with development stages based on shape features **(d)**, color features **(e)**, all features **(f)**. In a, b, and c, berry images obtained from different varieties using High-Throughput Phenotyping (HTP) tools are represented. Ellipses of different colors depict the confidence intervals for each cultivar. In d, e, and f, different symbols indicate distinct genotypes, while varying colors represent developmental stages: BV (Before Veraison), V (Veraison), AV (After Veraison), and R (Ripening).

**Figure S8.** Effects of different camera types **(a)** and image resolutions **(b)** on shape and color extraction values. Camera 1, SLR; camera 2, Huawei P40; camera 3, iPhone 6s; camera 4, Sony α6400, camera 5, Redmi K40 pro. The importance of the pedicel in determining the length and width of grape berry for different shapes **(c)**. Effect of angle between pedicel and the plate on image shape analysis **(d)**.

**Figure S9. Robustness of imaging and extraction for measuring phenotypes of berries in different orientations.** The raw images **(a, c, e)** and pedicel identification images **(b, d, f)** of berries in different orientations. Three different ways of positioning ten berries: berries in a line **(a, b)**, berries in a circle with pedicels inward **(c, d)**, and berries in a circle with pedicels outward **(e, f)**. The correlation between manually measured values and extracted values of shape traits **(g,** length: mm, **h,** width: mm**)** and color traits **(i,** color channel a, **j,** color channel b**)**. The error bars on the x-axis indicate the error of manual measuring done by three technicians.

**Figure S10. High-throughput phenotyping and association analysis of berry color.** The mean and intra-variation (sd) of berry color traits were phenotyped for 100 berries per genotype at maturity from the BF × Y73 population. PCA analyses were conducted to discriminate genotypes of the population with berry color traits. **a**, The distribution of PC1 for each genotype of the population is shown. **b**, Images of berries with extreme phenotypes (accession numbers indicated) according to PCA were projected into the PCA scattering space. **c**, The density profile of significant SNPs identified by GWAS for mean, intra-variation (sd), and composite (PCs) traits. The concentric circles from outer to inner show gene density (i), SNP density (ii), mean-based traits SNP density (iii), intra-variation (sd)-based traits SNP density (iv), composite (PCs) traits SNP density (v). Different color scale bars indicate the range of each density. The reference genome is PN40024 12X.v0. **d**, Venn diagram showing number of genes identified by mean, intra-variation (sd), and composite (PC) traits. **e-j**, Manhattan plots for the composite trait (PC1) from the high-throughput phenotyping of berry colors in 2020 (**e**), 2021 (**f**) and 2022 (**g**) and total anthocyanin of berry skin by HPLC in 2011 (**h**), 2012 (**i**), 2013 (**j**). The y-axis of the Manhattan plot represents the -log10 *P* value for candidate association using the GLM method. The vertical red line through the plots indicates the gene locus of the well-known berry color-related gene *MYBA1*.

**Figure S11.** Distribution of glucose, fructose, and total sugars content (g/L), contributing to the phenotypes of the BH × ES F_1_ population (183 samples) in 2011, 2012, and 2013. The normality of these soluble sugars was performed using the Shapiro-Wilk test, and the corresponding p-values are presented.

**Figure S12.** The raw distribution of tartaric acid, malic acid, and total acid content (g/L) that contribute to the phenotype of the BH × ES F_1_ population (183 samples) in the years 2011, 2012, and 2013 was examined. To assess the normality of these organic acids, the Shapiro-Wilk test was conducted, and the resulting p-values are presented.

**Figure S13. Genetic basis of soluble sugar. a-g,** Manhattan plots of GWAS results for soluble sugars in the BH × ES F_1_ population across multiple growing seasons. The GWAS analysis was performed using GLM Model in TASSEL 5.0. Negative log_10_(*p*) values from a genome-wide scan are plotted against the position on each of the 19 chromosomes. For glucose, fructose, and total sugar, significant loci were identified in three consecutive growing seasons, including 2011 2012, and 2013. The mapping was conducted using 183 individuals from the BH × ES F_1_ population. The red rectangle indicates a QTL that has been detected multiple times. The gray solid line represents the significant threshold, while the black dashed line represents the highly significant threshold. **h,** Gene ontology analysis of genes within the *TS1* locus. **i-j,** Gene structure, the major different genotypes of and boxplots for total sugar in 2011 based on the genotypes of *VIT_213s0019g00740* from haplotype analysis. **k,** Dot plots show the allelic combinations for total sugar in 2011. Black dots represent the progeny categorized according to different allelic combinations. The red dot indicates the medians for each category. The purple and green dots indicate the alleles of the parents. NA indicates progeny that do not carry any favorable alleles. The presence of different letters above the boxplots in **g**, **k** indicates statistically significant differences (p < 0.05) in the distribution of values among different genotypes, as determined through the application of Tukey’s test. The sample size was shown under each boxplot in **g** and **k**.

**Figure S14. QTL mapping results for soluble sugars content were obtained in the BH × ES population for the years 2011, 2012, and 2013, focusing on sucrose, fructose and total sugar content in mature berries.** The mapping was conducted using 183 individuals from the BH × ES F_1_ population. The red dotted line indicates the LOD threshold obtained from 1000 permutation trials, with a significance level of 0.05. The red dashed line represents the LOD thresholds, which were determined by 1000 permutation tests, with a significance level of 0.01.

**Figure S15. QTL mapping results for organic acid content of berries, including tartaric acid, malic acid and total acid were obtained in the BH × ES population for the years 2011, 2012, 2013.** The mapping was conducted using 183 individuals from the BH × ES F_1_ population. The red dotted line indicates the LOD threshold obtained from 1000 permutation trials, with a significance level of 0.05. The red dashed line represents the LOD thresholds obtained from 1000 permutation tests, with a significance level of 0.01.

**Figure S16. Gene ontology analysis and Dot plots of berry content of total acid.** **a,** Gene ontology analysis of genes within the TA1 locus. **b-d,** Dot plots of berry content of total acid in 2011 **(b)**, 2012 **(c)** and 2013 **(d)** in genotypes (black dots) according to different allelic combinations of *TA1* and *TA2* loci. The red dot indicates the median for each category. The purple and green dots indicate the acid content of berries with parental alleles. NA indicates progeny that do not carry either high-acid allele. The presence of different letters above the boxplots in **b**-**d** indicates statistically significant differences (p < 0.05) in the distribution of values among different genotypes, as determined through the application of Tukey’s test. The sample size was shown under each boxplot in **b**-**d**.

**Figure S17. The effects of allelic combinations for variation in the proportion of total berry sugar and total berry acid in the population.** Each circle represents an individual. Circle size indicates the allelic combination for total sugar in that individual. Circle color indicates allelic combinations for total acid. Favorable markers are *TS1* and *TS2* for total sugar and *TA1* and *TA2* for total acid, respectively. NA indicates individuals carrying no favorable alleles. The solid blue line indicates the linear regression line fitted to the data, representing a significant linear regression model (p < 0.05) with a sample size of 237. The variation in sugars and total acids of each individual was calculated in comparison with the average value of all the individuals in the BH x ES population, with positive values for individuals higher than the population average and negative values for individuals lower than the population average.

**Figure S18. Phenotypic distributions and correlations of winter bud cold tolerance evaluated with low-temperature exotherms (LTE) over six growing seasons.** Distributions of LTE in the individuals during the growing seasons of 2012, 2013, 2014, 2015, and 2016 in the F_1_ BH × ES population (165 samples), as well as in germplasm population in 2018 (118 samples). To assess the normality of the distributions, the Shapiro-Wilk test was conducted, and the resulting p-values are presented.

**Figure S19.** The Manhattan and QQ plots display the GWAS results for LTE in the F_1_ BH × ES population (165 samples) in five growing seasons, as well as in the germplasm resource population (118 samples) for the year 2018 **(f)**. The vertical axis represents the negative log_10_(*P*) values, while the horizontal axis represents the position on each of the 19 chromosomes. The GWAS analysis was performed using GLM Model in TASSEL 5.0. Significant loci were identified in 2012 **(a)**, 2014 **(c)**, 2015 **(d)**, and 2016 **(e)**. The **(c)** pie charts represent allelic frequencies of *LTE1* and *LTE2* loci in the GWAS population, and the blue portion represents low LTE values, while the red portion represents high LTE values. Only the locus *LTE1* on chromosome 3, highlighted with a red rectangle of dashed lines, was detected in two growing seasons (2012 and 2015). In 2018, a candidate gene, *NAC08*, was identified within the *LTE3* locus, which also overlapped with QTL mapping results from the F_1_ BH × ES population (Figure 6). The grey solid line represents the significant threshold, while the black dashed line represents the highly significant threshold of p-values **(a-e)**. The red line shows the highly significant threshold for p-values **(f).**

**Figure S20.** Expression pattern of *NAC08* under cold treatment was conducted in *V. amurensis* and ‘Muscat Hamburg’. The different letters above the bars indicate that the values among the group are significantly different (p < 0.05), as determined by the Tukey’s test based on three biological replicates.

**Figure S21.** The *NAC08* coding sequences of *V. amurensis* (*VaNAC08*) and ‘Muscat Hamburg’ (*VvNAC08*) were compared by aligning the sequences using DNAMAN.

**Figure S22.** A comparison of amino acid sequences between *V. amurensis* (*VaNAC08*) and ‘Muscat Hamburg’ (*VvNAC08*) was conducted using DNAMAN for alignment.

**Figure S23. Verification of the insertion and expression of *VaNAC08* in the overexpressing calli was performed**. **a,** The insertion of *VaNAC08* and NPTII was detected. **b*,*** The expression of *VaNAC08* was assessed, with Actin and MDH serving as internal controls. Different letters above the bars indicate significant differences among the groups (p < 0.05), as determined by the Tukey’s test based on three biological replicates.

**Figure S24. Knockout of *NAC08* in grape calli was successfully achieved through the precise targeting specific sites in the coding sequences using CRISPR/Cas9 technology. a**, The schematic diagram illustrates the target sites within the coding sequence, denoted as T. Primers SP-F and SP-R were utilized for PCR amplification. **b,** Two example chromatograms showing a microdeletion, as well as representative sequences with insertion mutations and deletion mutations identified from **(d)**. **c-d,** The sequencing results of EV **(c)** and mutations **(d)** were identified from 20 clonal amplicons of calli. The homologous nucleotides are shaded, with different colors indicating varying levels of homology. Nucleotides with a homology level of 100% are shaded in blue, and those with a homology level of ≥75% are shaded in red. Red numbers on the right indicate the number of detected clones with the same mutation type. **e,** Mutations of amino acids in the corresponding mutated sequences in **(d)**.

**Figure S25. Overexpression of VaNAC08 improves cold tolerance in 4-week-old Arabidopsis plants. a,** Freezing phenotypes of the OE lines (OE1, OE2) and WT control. **b,** Survival rate of transgenic Arabidopsis after freezing treatment (-8 ℃ for 8 h, 4℃ for 12h). **c,** Electrolyte leakage of OE lines and WT plants after freezing treatment. The error bars indicate the SD from triplicate technical repeats. Different letters above the bars indicate significant differences based on three biological replicates (p < 0.05, Tukey’s test).

**Figure S26. Verification was performed on the insertion and expression of *VaRFS6* in the overexpressing calli**. **a,** Detection of *NPTII* insertion was carried out. **b*,*** Evaluation of *VaRFS6* expression was performed, with Actin and MDH serving as internal controls. Significant differences among the groups were indicated by different letters above the bars (p < 0.05), as determined by the Tukey’s test based on three biological replicates.

**Figure S27. Knockout of *RFS6* in grape calli was successfully achieved through precise targeting of specific sites in the coding sequences using CRISPR/Cas9 technology. a**, the schematic diagram illustrates the target sites within the coding sequence, denoted as T1, T2, with primers SP-F and SP-R utilized for PCR amplification. **b,** two examples chromatograms show a microdeletion, as well as representative sequences with insertion mutations and deletion mutations identified from sequencing results. **c,** the mutations identified from 20 clonal amplicons of calli show varying levels of homology, with homologous nucleotides shaded in different colors indicating the level of homology. Nucleotides with a homology level of 100% are shaded in blue, and those with a homology level of ≥75% are shaded in red. Red numbers on the right indicate the number of detected clones with the same mutation type. **d,** Amino acid mutations in the corresponding mutated sequences were also identified.

**Figure S28. The model of VaNAC08 TF action during grape cold tolerance identified in this study.** It suggests that the upregulation of VaNAC08 expression under cold tolerance conditions leads to the activation of *VaRFS6*, resulting in increased raffinose accumulation. This, in turn, triggers the transcriptional activation of stress-inducible genes, ultimately enhancing the cold tolerance of grapevine.

**Figure S29. Distribution and QQ plots for the flower sex.** Distribution of hermaphrodite and female individuals in BF ×Y73, BC × CoC and BH × ES populations **(a)**. QQ plots of the GWAS analysis for the flower sex, performed using GLM in TASSEL 5.0, based on 454 *Vitis* pooled genotypes from three breeding populations, and separately for the F_1_ genotypes of the BH × ES, BF × Y73, and BC × CoC populations **(b)**.

**Figure S30. Networks representing the relationships between QTLs and different traits.** QTLs are associated with shape **(a)** and color traits **(b)** from HTP tool. Additionally, traits measured through conventional methods are divided into different groups, along with soluble sugars, organic acids, LTE (low temperature exotherms), and flower sex **(c)**. The red color in figure **(c)** represents the LOD (likelihood of odd) value in QTL mapping or the p-value in GWAS. The circle represents the trait of each year, and the diamond represents the QTL locus **(c)**.

**Figure S31. Loci linking with multiple traits.** The blocks within the chromosome represent the positions of QTLs, and the color of the blocks indicates the frequency of loci identified by GWAS and QTL mapping across all traits. Symbols represent loci identification methods, including QTL mapping (triangle) and GWAS (circle). Colors outside the chromosome represent trait categories. The detailed list of QTLs is shown in Table S26, Supporting Information.

**Figure S32. Relatedness to parents for the three F_1_ breeding populations, comprising a total of 455 genotypes.** Relatedness to parents in the F_1_ family of BF × Y73 (**a**), BH × ES (**b**), and BC × CoC (**c**). One to six individuals with weaker relatedness to parents as a result of self-pollination, outcross, or mislabeling were identified and excluded from the following genetic analysis.

**Supporting Information**

**Table S1.** Summary of grapevine accessions used for SNP identification and for designing the 200K Axiom® SNP array

**Table S2.** The annotation of the 174464 SNPs

**Table S3.** The genomic positions on 19 chromosomes of 2K rhAmpseq, 18K SNP chip, and 200K Axiom® SNP array

**Table S4.** The 2K rhAmpseq, 200K Axiom® SNP array, and 18K SNP chip cover different sets of genes

**Table S5.** Summary of grapevine accessions genotyped by the 200K Axiom® SNP array for validating the array

**Table S6.** Genotyping results of six SNP types

**Table S7.** The genetic maps of three populations

**Table S8.** Summary of the nineteen linkage groups in the three F1 populations

**Table S9.** Tool extracted traits, symbols, fruit trait names, description, and explanations

**Table S10.** Comparison of conventional methods with high-throughput phenotyping

**Table S11.** QTL and QTN results of individual color traits composite color traits shape traits and composite shape traits

**Table S12.** All candidate genes about *Shape1* identified by association mapping

**Table S13.** List of significant SNPs associated with soluble sugars, organic acids, LTE, and flower sex identified by GWAS

**Table S14.** The association mapping results of soluble sugars

**Table S15.** All candidate genes within *TS1* related to soluble sugars

**Table S16.** All candidate genes within *TA1* related to organic acids

**Table S17.** Summary of 101 grapevine accessions in gerplasm resources population

**Table S18.** The association mapping results of cold tolerance

**Table S19.** All candidate genes within *LTE1* related to cold tolerance

**Table S20.** Transcriptomic data analysis reveals correlation of genes with *NAC08*

**Table S21.** Summary of transcriptomic data and upregulated expression results of VaNAC08-OE compared to the empty vector under normal temperature conditions

**Table S22.** GO enrichment analysis of the 399 overlapping genes that exhibit a strong correlation with NAC08 (Table S20) and show upregulated expression in VaNAC08-OE compared to the empty vector (Table S21)

**Table S23.** The downstream candidate target genes of NAC08 involved in the term of 'responding to stimulus' in Table S22.

**Table S24.** The χ² and p-value of flower sex in three populations

**Table S25.** The association mapping results of flower sex

**Table S26.** List of QTL loci associated with color, shape, size, soluble sugar, organic acid, LTE, and flower sex identified by GWAS and QTL mapping
